# Developmental Differentiation and Binding of Mental Processes with *g* through the Life-Span

**DOI:** 10.3390/jintelligence5020023

**Published:** 2017-05-31

**Authors:** Andreas Demetriou, George Spanoudis, Smaragda Kazi, Antigoni Mouyi, Mislav Stjepan Žebec, Elena Kazali, Hudson Golino, Karin Bakracevic, Michael Shayer

**Affiliations:** 1 Department of Social Sciences, University of Nicosia, 1700 Nicosia, Cyprus; 2 Department of Psychology, University of Cyprus, 1678 Nicosia, Cyprus; spanoud@ucy.ac.cy (G.S.); antigoni.mouyi@gmail.com (A.M.); 3 Department of Psychology, Panteion University of Social Sciences, 176 71 Athens, Greece; smakazi@otenet.gr (S.K.); elenakazali@hotmail.com (E.K.); 4 Department of Psychology, Croatian Studies University of Zagreb, 10000 Zagreb, Croatia; mzebec@hrstud.hr; 5 Department of Psychology, University of Virginia, Charlottesville, 22903 VA, USA; hfgolino@gmail.com; 6 Department of Psychology, University of Maribor, 2000 Maribor, Slovenia; karin.bakracevic@um.si; 7 King’s College, University of London, London WC2R 2LS, UK; m.shayer@btinternet.com

**Keywords:** intelligence, cognitive development, individual differences, integration/differentiation, awareness

## Abstract

Integration/differentiation of mental processes is major mechanism of development. Developmental theories ascribe intellectual development to it. In psychometric theory, Spearman’s law of diminishing returns postulates that increasing *g* allows increasing differentiation of cognitive abilities, because increased mental power allows variable investment in domain-specific learning. Empirical evidence has been inconsistent so far, with some studies supporting and others contradicting this mechanism. This state of affairs is due to a developmental phenomenon: Both differentiation and strengthening of relations between specific processes and *g* may happen but these changes are phase-specific and ability-specific, depending upon the developmental priorities in the formation of *g* in each phase. We present eight studies covering the age span from 4 to 85 years in support of this phenomenon. Using new powerful modeling methods we showed that differentiation and binding of mental processes in *g* occurs in cycles. Specific processes intertwine with *g* at the beginning of cycles when they are integrated into it; when well established, these processes may vary with increasing *g*, reflecting its higher flexibility. Representational knowledge, inductive inference and awareness of it, and grasp of logical constraints framing inference are the major markers of *g*, first intertwining with in their respective cycles and differentiating later during the periods of 2–6, 7–11, and 11–20 years, respectively. The implications of these findings for an overarching cognitive developmental/differential theory of human mind are discussed.

## 1. Introduction

More than a half a century ago, Lee Cronbach [1] set a major goal for psychology: To integrate experimental psychology, which studies lawful relations between processes, with correlational psychology, which studies individual differences in processes, into a unified discipline that would allow predictions of “the behavior of organism-in-situation” (p. 682). Although the goal is still valid, considerable progress has been achieved since then. In the domain of the sciences of the human mind, age differences and within-age individual differences in intelligence are systematically connected to the state and operation of several mental processes. These relations will be outlined below, although it is beyond the aims of this article to exhaustively review or evaluate this research (see [2,3,4,5]). Instead, we will build on what seems to be common ground in order to highlight how processes interact in development, causing deep changes in how the human mind operates in different phases of life. Here we present research covering changes from the age of 4 to 85 years.

“Altogether, *g* is a search and align, cognize and choose, and abstract and reason mechanism. Notably, each of the processes involved (executive control, flexibility, working memory, cognizance, and inference), at any age, are autonomous and distinct contributors to *g*.” [6]. They are all always part of the mind because they are somehow dependent on each other. Executive control requires awareness of a goal, the steps needed to obtain it, and an inference-based decision system allowing evaluation of goal attainment. Reasoning requires awareness of gaps in information (yielding a reasoning goal), of the representations to be integrated, and of truth standards needed to evaluate conclusions. Cognizance, awareness of cognitive processes, is a context-manipulating mechanism allowing interfacing and holding executive control and reasoning together to collaborate. The interaction between cognizance and reasoning allows metarepresentation which encodes similarities between representations into new representations, thereby driving intellectual development.

“What varies is their relative contribution with development. Overall, the contribution of the executive processes diminishes and the contribution of the cognizance and inference increases with age as the first automate and the second are increasingly needed to handle the increasing multiplicity of the representations mediating between the individual and the world.” [7]. The studies to be presented below were designed to capture how the relations between specific mental processes and *g* change with development. In particular, this article focuses on a long-debated issue in the psychology of intellectual development and individual differences: Are special mental processes differentiated from or integrated with general mental ability as general mental ability or age increases? We present a series of studies answering this question for a succession of developmental periods spanning from 4 through 85 years of age. 

## 2. The Cycles of Intellectual Development

Research suggests that the relations between these processes are transformed over four major developmental cycles, with two phases in each. New representations emerge early in each cycle and their alignment and integration dominates later (see [7] for details). In succession, the four cycles operate with *episodic representations* from birth to 2 years (remembrances of actions and experiences preserving their spatial and time properties), *mentations*, i.e., *realistic mental representations* from 2 to 6 years (blueprints of episodic representations where spatial and time properties are reduced, associated with symbols, such as words), *generic rules organizing representations* into conceptual/action systems from 6 to 11 years, (e.g., concepts about categories of things, exploring causal relations) and lastly *overarching principles integrating rules* into systems where truth and multiple relations can be evaluated from 11 to 18 years (i.e., principles specifying how rules may be integrated). Changes within cycles occur at 4, 8, and 14 years, when representations become explicitly cognized so that their relations can be worked out, gradually resulting into representations of the next cycle [7]. 

The central assumption, from the point of view of development, is that control of action provides the primary material one may become aware of. Becoming aware of one’s own action allows considering choices of goals, plans, and alternative actions in sake of success and efficiency. Reasoning is a means to this end, which allows evaluating odds, truth, and validity in sake of efficient action. In any cycle, awareness of control experiences is transformed into models of the mind that may be called upon in sake of reasoning. Thus, changes in control at the beginning of cycles are expressed into explicit executive control plans in the middle of the cycle and these are then expressed into complex reasoning-inferential systems at the end of the cycle. When metarepresented into new inferential schemes the next cycle begins. Below we focus on all cycles but the first because this article presents studies addressed to these cycles. 

Two points are in order here. On the one hand, age boundaries are modal idealized specifications rather than fixed points of change in every individual. They reflect major changes in the majority of individuals in how the world is represented but there may be large individual differences in the exact age of moving across phases or cycles. On the other hand, earlier types of representations do not vanish when a transition to a later kind occurs. They are there and they may be habitually used if context allows. However, when achieved, a new type of representation imposes an urge to organize information and its processing in ways appropriate for its level. For instance, rule-based representations orient the thinker to look for links between iconic representations and their underlying inferential threads. Principle-based representations orient the thinker to look for overarching value-weighted rule packages. The dominant type of representation in a cycle operates as an advanced organizer for incoming information and an organizational registry for information already processed. As such, they generate representational urges, so to speak, which have a strong subjective aspect in how one relates to the world and a psychological procedural aspect in how one must deal with the world.

### 2.1. Realistic Representational Thought

Representations at 2–3 years of age are reduced mental projections of episodic representations with a component of implicit awareness. Children at 3.5 years implicitly differentiate between correctly remembered (e.g., objects observed carefully) and not remembered items (e.g., objects seeing only for a little), suggesting an awareness of representations stored earlier in memory [8]. Also, toddlers are aware that when one saw an object one knows about it [9]. This makes Theory of Mind (ToM) possible, enabling toddlers to understand that one’s actions relate to one’s representations [10].

By the age of 4–5 years executive control is guided by a “focus-recognize-respond” program allowing toddlers to set-up action plans requiring shifting between actions according to a probe. For instance, “Sort according to color when a red tag is on and according to shape when a square tag is on” (see [11]). This task involves awareness of representations one may focus on and choose from, organizing action beforehand. By this age, reasoning ascents from reciting or reading episodic blocks forward to pragmatic deals: “You said I can play outside if I eat my food; I ate my food; I go to play outside.” [12]. This sequence, is basically an inference locking two representations (“A occurs” and “B occurs”) together into an inductive sequence (i.e., When A occurs, B also occurs). 

### 2.2. Rule-Based Thought

At 6–8 years, children are explicitly aware of mental representations and their relations with their own actions. For instance, they differentiate between easy and difficult memorization tasks, suggesting awareness of the relation between complexities of representations and learning [8]. However, at this age, children do not yet explicitly differentiate between mental functions, such as memory and reasoning, nor do they explicitly associate each with specific processes (rehearsal vs. inference). This is possible at 8–10 years [13], when there is an explosion of awareness of the mental world. Children in this phase master second-order ToM (e.g., “I know that George knows that Mary knows that …”; [10], recognize that gaps in knowledge may be compensated by inference (e.g., He sorted by color, so blue objects would be in the blue box; [14]). 

In the next phase, at 8–9 years, awareness of different mental processes allows children to shift flexibly between them (e.g., to remember you need to observe carefully and rehearse; to sort you need to follow a sorting rule; [15,16]). Thus, in this phase, executive control is upgraded into a *conceptual fluency program* allowing children to shift between mental processes (e.g., memory vs. inference) or conceptual domains (e.g., they recall words belonging to different categories—fruits, animals, furniture—following a probe; [17]). Compared to the previous “focus-recognize-respond” executive program, the current program involves analytic representations of conceptual spaces.

Also, they implicitly use rules specifying how different types of inference are interrelated. For instance, if accepted that “A implies B” then two possibilities are necessarily true: When A occurs then B occurs too and when B does not occur then A did not occur either [18]. Therefore, awareness of underlying relations allows moving across conceptual spaces and rules that they may then guide executive control and reasoning. Grasp of logical necessity in this phase is a strong sign of this awareness (e.g., All balls in the box are red, so the next to be drawn out MUST be red; [19]). 

### 2.3. Principle-Based Thought 

At 11–13 years, adolescents form accurate maps of mental functions and of their own strengths and weaknesses [20,21,22]. Thus, they cognize the constraints of different inferential processes and they can ground inference on truth and validity rules. That is, they explicitly understand that accepting certain conditions (e.g., birds fly; elephants are birds) imposes constraints on inference (i.e., elephants fly) even if a statement is admittedly wrong (elephants are not birds). This achievement allows consistency in reasoning. By the age of 13–14 years, “reasoners have a meta-representation of logical validity that can be used to inform them of the accuracy of their logical deductions, at least when reasoning about abstract materials.” [23] (p. 691). This protects them from drawing false conclusions. Specifically, they understand that accepting that “If A then B” does not allow drawing any conclusion about A if only knowing that B occurred or drawing any conclusion about B if only knowing that A did not occur because B may be caused by causes other than A. Therefore, the inferential relevance mastery program explicitly places truth weights on the various alternative choices that may be deduced from a logical argument [18,24]. Executive control in this cycle is very different from the previous cycles. It is based on a suppositional-generative program enabling adolescents to co-activate conceptual spaces and evaluate them against each other [25].

## 3. The Integration/Differentiation Dispute

Psychometric theory and developmental theory agree that mental possibilities change with growth. Mental age in psychometric theory and stage in developmental theory both capture enhancement of mental ability with age. They both indicate that individuals deal with concepts and problems of increasing abstraction and complexity as they grow. Theories also agree that individuals differ in rate of enhancement or final attainment. IQ in psychometric theory recognizes that chronological and mental age may not coincide and specifies how they relate in an individual in reference to this individual’s age group. Developmental theory considers stages as ideal epistemic states corresponding to successive age periods and recognizes that rate of progression along stage sequences or final stage attainment may differ across individuals. Thus, differential psychology examines cognitive development through the lens of individual differences while developmental psychology focuses on developmental differences. 

Several mechanisms were invoked to account for developmental progression and ensuing enhancement of mental ability with age. The twin mechanism of integration/differentiation of mental processes is a major mechanism of development. Cognitive developmental theories postulate that increasing ability comes from increasing integration/differentiation of mental processes [26,27,28]. Piaget’s equilibration is a developmental mechanism generating increasingly coordinated (integrated) mental structures that become increasingly precise in their environmental implementation because they can efficiently accommodate (differentiate) concepts and problems and operate accordingly. In psychometric theory, ability-differentiation refers to variation in actual abilities that goes with increasing general intelligence. Specifically, psychometric theory postulates that increasing *g* allows increasing differentiation of cognitive abilities, because there is expansion of mental power which may be invested into domain-specific learning, causing domains to differentiate. This is Spearman’s law of diminishing returns for age (SLODRage) [4,29]. The developmental adaptation of Spearman’s differentiation hypothesis would assume that abilities differentiate with growth because *g* increases with development. 

Technically, decrease of correlations between abilities with increasing *g* was considered as evidence favoring differentiation. In terms of factor analysis, the equivalent would be an increase in the number of factors needed to account for performance of high *g* individuals as compared to lower *g* individuals or a decrease in the between-factor correlations in oblique factor models. According to Detterman’s [30] systems theory of mental retardation, the malfunctioning of central mechanisms in individuals with low intelligence causes homogeneously lower performance across abilities: Hence higher correlations and stronger *g*. Recent research provided rather weak and inconsistent evidence probably because increases in ability and age go partly together thereby confounding each other. Specifically, some studies did find the expected pattern of decreasing correlations or increasing number of factors with increasing age (e.g., [25,31,32,33]) but others did not [34]. Others found that ability differentiation is age dependent, in the sense that it shows up only in late childhood [35]. Using methods allowing separation of ability from age, several researchers found clear evidence in favor of ability differentiation but not in favor of age differentiation [36,37,38]. Obviously, this finding aligns with the distinction between mental and chronological age, implying that differentiation of abilities occurs as a function of mental rather than chronological age. Interestingly, Carroll [39] himself, analyzing several data bases in search of evidence for differentiation, concluded “that this is a phenomenon whose evidence is hard to demonstrate. … The question of age differentiation is probably of little scientific interest except possibly at very young ages. It is, if anything, of more scientific interest that the same factors are found throughout the life span.” (p. 681). Realistic as it might sound, this interpretation begs the question of the mechanism underlying systematic changes and/or differences in mental age. 

The present model offers a reason for this state of affairs. Specifically, this model suggests that differentiation may vary according to developmental phase and/or the dominant representational characteristics of *g*. That is, both differentiation and strengthening of relations between specific mental processes and *g* may happen but these changes in the relations between *g* and specific abilities are phase-specific and ability-specific, depending upon the developmental priorities in the formation of *g* in each phase. Specifically, the primary developmental task in the cycle of realistic representational thought is representational control. This may express itself in several forms: Control of attention focus, control of shifting between stimuli and/or responses, linking an action sequence to a plan (e.g., puzzle completion). The primary developmental task of the next cycle of rule-based thought is inferential control. That is, the command of the inferential process so that it can fill in gaps of information systematically. This is primarily expressed through changes in awareness of the inferential processes itself and also through improvements in the application of the inferential processes as implicated in analogical and Raven-like tasks. The primary developmental task in the cycle of principle-based thought is command of cognizance and related rules so as to ensure truth and validity of inference. This is primarily expressed through accuracy in self-representation and self-evaluation. Therefore, mastering (i) attention control and mental representation; (ii) rule-based relations and awareness of underlying inference; and (iii) principle-based relations and awareness of related processes must get increasingly connected to *g* in the cycle of realistic representational, rule-based, and principled-based thought, respectively. Each of these processes ought to spurt in the developmental phase concerned (i.e., 4–6, 6–8, and 11–13 years, respectively), marking the major acquisition of *g* in each phase.

## 4. Methods

### 4.1. The Differentiation Model

**Figure 1:**
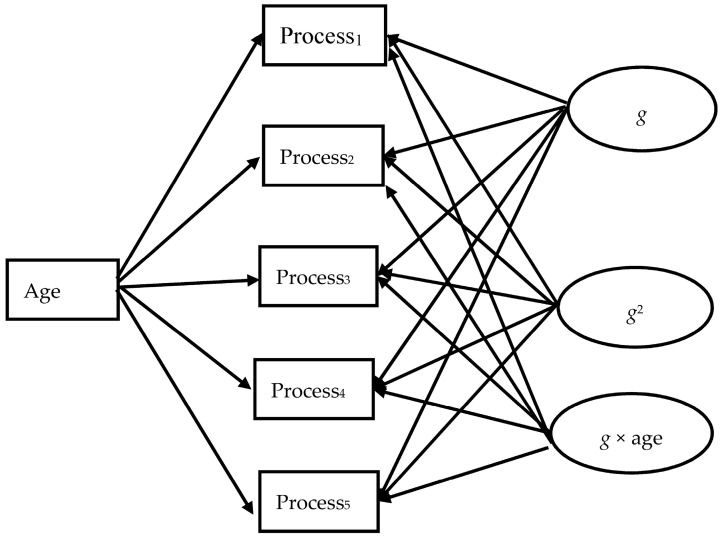
The general model for testing possible differentiation of mental processes from general intelligence (*g*). Note: Each process is regressed on age, a common factor (*g*), quadratic *g* standing for ability differentiation, and the *g* × age product, standing for age differentiation.

To examine the ability/age differentiation hypothesis, a model recently proposed by Tucker-Drob [36,38] was employed. This is a structural equation model allowing testing the possible differentiation of abilities with increasing *g* and/or age. This model, illustrated in Figure 1, specifies how abilities relate to *g*, age, a factor standing for possible differentiation of abilities from *g* according to increasing *g*, and a factor standing for a possible differentiation of abilities as a function of age.

Technically, a standardized measure of each ability is regressed on a common factor standing for *g*, on age, on quadratic *g*, and on the age × *g* product to stand for the relations specified above, respectively. Quadratic *g* is considered to be an *ability differentiation index* because squaring *g* maximizes possible effects at the higher end of *g*. This can capture if a specific ability becomes inflated at higher levels of *g*. In the same fashion, the *g* × age product is an index of *developmental g* maximizing the possible effect that the interaction between *g* and age might have at higher levels of ability and/or *g* on specific abilities, if present. It is noted that one might use the quadratic effect of age rather than the *g* × age interaction to capture developmental *g* [36,38]. This possibility was examined and rejected in a series of preliminary analyses applied on each of the studies following. Using quadratic age rather actual age did not account for any additional variance in the models, probably because the age windows used here were rather limited, due to our focus on the age phases of interest.

To examine if each factor is needed to account for performance on the various processes, the model is tested in a stepwise fashion: That is, at a first run, the ability-specific indexes used in the model above are regressed on *g* and age. A second run involves two alternative models: (i) in the first, quadratic *g*, the ability differentiation index, is also included in each equation; (ii) in the second, quadratic *g* is dropped and the *g* × age product, the age differentiation index, is used. Finally, all factors are included in the model. A construct is needed if the model where it is involved fits better than a simpler model not including it.

The direction of relations is also important: Specifically, “it is important to inspect the direction and statistical significance of each of the terms in order to evaluate whether the ability differentiation and age differentiation hypotheses were supported. To accept such support, the parameters should be in directions indicative of lower loadings at high ability levels, [and] lower loadings with increasing childhood age … Moreover, the effects should not be isolated to a single broad ability, but should instead be statistically significant and consistent in direction for multiple abilities.” [38] (p. 1107). Specifically, differentiation is indicated by negative relations between a process and a differentiation factor. A positive relation would indicate that a process gets increasingly intertwined with increases in the factor concerned, such as *g* or age × *g*. Based on the model outlined in the introduction, we expect that the abilities dominating in each cycle as major contributors to *g* would intertwine with rather than differentiate from *g*. For instance, the relations between (i) attention control; (ii) inferential awareness and inductive inference; and (iii) awareness of specific processes and advanced deductive reasoning with *g* would strengthen in the cycle of realistic representations, rule-based, and principle-based thought. Each of these processes may differentiate from *g* in the cycles following, reflecting that they are not part of the priorities of *g* formation any more. 

Attention is drawn to the scores used in the various models. Whenever possible, performance on reasoning batteries and many of the awareness measures was subjected to Rasch analysis and the logit score indicating each individual’s ability on each battery was used. This was not possible for reaction time measures, working memory, and some of the awareness measures. However, to ensure comparability of measures as much as possible, these measures were converted to *z* scores in all studies. Reaction time scores were inverted in all studies so that their relations with age and the other scores vary in the same direction. Finally, age was always centralized (i.e., age—sample age mean). It is admitted that the multiplicity and the complexity of the studies involved necessitated a divergence from the ideal score formation as suggested by Tucker-Drob [38], where only reasoning tasks were involved. Thus, to the extent that this divergence may distort relations between processes, caution is needed in interpreting our findings. It is stressed, however, that the uniformity of approach adopted across all of the studies presented below allows answering the central questions of the study, that is, of relations between specific abilities and *g* vary with developmental phase.

### 4.2. The Developmental Model

Obviously, changes in the relations between each process and the differentiation factors suggest that the rate of change of each process varies with increases in ability and/or age. For instance, change in a particular mental process *M* accelerates after *g* reaches a particular level (partly associated with age) to match this level and it decelerates as it approaches this level. It is notable that the succession of these changes conforms to a logistic growth function that has been regarded by developmental theorists to capture the dynamics of change in several processes [40,41]. Specifically, the model of nonlinear logistic growth posits that change is very rapid at first, attaining maximum rate of change around the middle of its course, slowing afterwards as it approaches its final level, when a new cycle will start anew. The equation for the logistic growth curve is given by Equation (1) below:

(1) $$f\left(x\right)=\frac{l}{1+e^{-a\left(t-t_{0}\right)}}$$

**Figure 2:**
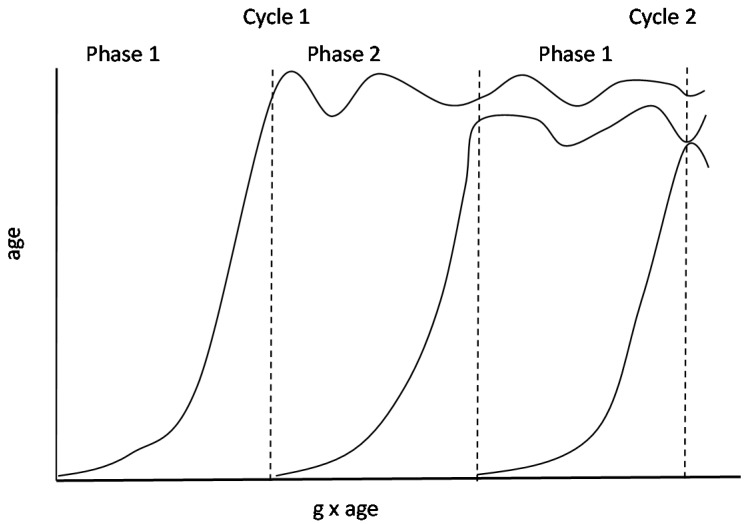
Idealized curves of the integrated integration–differentiation logistic growth model. Note: The Figure applies to development during the three cycles concerned; it does not model change after young adulthood.

In this equation *l* represents the peak of a developing ability or the final level of the ability at the end of the growth spurt, α is a parameter stretching or compressing time of development, quantifying the rate of change during the growth spurt, *t* and *t*_0_ symbolizes the age points at which the growth spurt begins and reaches a midpoint, respectively. As the distance between *t* and *t*_0_ becomes smaller approaching 0, the steeper the curve becomes, reflecting increased growth rate. It is predicted here that the variations in the relations between specific processes and *g* to be uncovered by the differentiation model above can only be understood if coupled with the fundamental non-linear logistic growth model. Specifically, the relation between a mental process M and *g* strengthens with decreasing distance between *t* and *t*_0_. After the crucial peak point, when development decelerates, the *M*–*g* relation gets looser, allowing differentiation. This alternation of tightening and loosening of *M*–*g* relations according to developmental phase is illustrated in Figure 2. 

To capture these changes we used segmented or piecewise linear regression. In the various models tested we always used the age differentiation factor (*g* × age) as the explanatory variable and the scores standing for each process as criterion variables. To obtain a person’s score on this factor, we first ran a factor analysis on the scores used in a study. These were always the scores used in the corresponding Tucker-Drob model tested on a study. In this type of analysis the first principal component captures what is common between all tasks. Thus, this is a good measure of psychometric *g*. The factor score of persons is an index showing how much of *g* a person possesses. This is actually something like a mean of the person’s performance on the tasks used weighted according to the relation of each task with the principal component. Each person’s factor score on the first principal component was multiplied with this person’s age. Obviously, this index is identical with *the age differentiation index* a specified in the Tucker-Drob model above. We opted for this index rather than the ability differentiation index because it includes the developmental dimension which is important both in our model and the logistic growth model. Therefore, this index stands for *developmental g* because it reflects both general ability and age.

We computed linear and segmented regression models for each process. We fit linear regression models of the form *y_i_* = *a* + *bx_i_* + *e_i_*. These models provide estimates of direction and rate of developmental change for abilities which do not show signs of qualitative transitions with development. However, segmented regression models allow estimating if there are break points in development that may be taken to stand for qualitative changes in development. Thus, these developmental models may highlight the developmental mechanism underlying differences between processes in their relation with changes in *g* and age, according to developmental cycle and phase. The *F*-test and Akaike information criterion (AIC) was used to decide if a linear or a segmented linear model best fits the age related patterning of performance on each criterion variable [42]. We will present exemplary models for each study to highlight how age and ability differentiation express changes in developmental progression.

We tested both the differentiation and the developmental model on the data of a series of studies covering, altogether, the age period from 4 to 85 years of age. All studies are published and therefore the reader is referred to the relevant publications for details. In the present context we will provide information about samples and task batteries that is necessary to follow the rationale and findings of each study. It is noted, however, that the present modeling is first presented here.

## 5. Results

### Study 1: Modeling an Exemplary Study

To demonstrate the value of these methods and their sensitivity to age variation, we model the data of the integrated sample first presented in [25]. This sample involved 662 participants about equally drawn from each of the age years 4 through 16. There was an index of five abilities for each participant: (i) Information processing speed, reflecting performance on the compatible version of Stroop like tasks (e.g., read color words written in the same ink color—the word GREEN written in green ink, say green) or Simon-like tasks where participants recognize the location of an object (left or right half the screen); (ii) attention control reflecting performance on incompatible Stroop-like tasks—recognize the ink color of a different meaning color word, e.g., the word RED written in green, say green; (iii) verbal working memory (recall of words in presentation order); (iv) visuo-spatial working memory (recall of shape, position, and orientation of geometric figures); (v) reasoning (a logit score standing for performance on verbal, quantitative, and spatial reasoning tasks systematically varying in developmental demand). The reasoning battery involved tasks addressing reasoning ability spanning from the second phase of realistic representations to the second phase of principled-based thought. Participants were examined by age-appropriate tasks so that their logit score indicates their standing on the developmental sequence described above. Correlations between the variables used in the various models are shown in Supplementary Table S1. 

The Tucker-Drob model was first applied on the whole sample. In this model, all five indexes of the processes involved were regressed on the factors specified above and shown in Figure 1 (i.e., age, *g*, quadratic *g*, and *g* × age). The results are summarized in Table 1. The columns of interest are those showing the relation between each process, quadratic *g*, and *g* × age. It can be seen that the relation between reasoning and quadratic *g* (−0.10) reached significance reflecting a weak differentiation of reasoning with increasing *g*. Interestingly, the relation of speed (−0.21) and verbal working memory (−0.20) with quadratic *g* was also negative, indicating their differentiation with increasing *g*. The relation of speed (0.36) and control (0.32) with age differentiation was highly significant and positive, suggesting that these two processes intertwined (improved) rather than differentiated with increasing age. Notably, all correlations between the variables included in the model were highly significant, and ranged between moderate and very high (i.e., >0.7). 

The picture is quite different when the model is tested on different age periods. Specifically, the model was tested separately in three groups (4–6-year olds, *N* = 112; 7–10-year olds, *N* = 300; and 11–17-year olds, *N* = 250). It can be seen that in the younger age phase, the relation of speed (3.04), attention control (3.00) and working memory (0.30) with the age differentiation index was high and positive. This pattern indicates that attentional and visual working memory processes build up with age in this phase, powerfully contributing to the formation of *g*. It is notable, however, that both speed (−3.19) and control (−3.10) were highly and negatively related to the ability differentiation index, indicating higher variation of response times at higher *g* levels in this age phase. The pattern is very different in the 7–10 years phase. In this phase both speed (−1.16 and −0.84) and control (−0.89 and −0.26) did differentiate highly from both ability and age. However, in this phase, the relation of reasoning (0.12), verbal (0.49) and visual working memory (0.22) with the age differentiation index was significant and positive, indicating that command of the inferential processes and the symbolic processes related to working memory are the main contributors to the formation of *g*. In the 11–17 years phase the pattern changed again. Specifically, speed and control intertwined (improved) with *g* (0.55 and 0.34, respectively) but differentiated with age (−0.87 and −0.53, respectively) and visual working memory differentiated from ability (−0.25) but intertwined with age (0.28). Reasoning and verbal working memory did not relate with any differentiation index. Overall then, changes in processing efficiency go together with linear changes in reasoning and verbal working memory.

**Figure 3:**
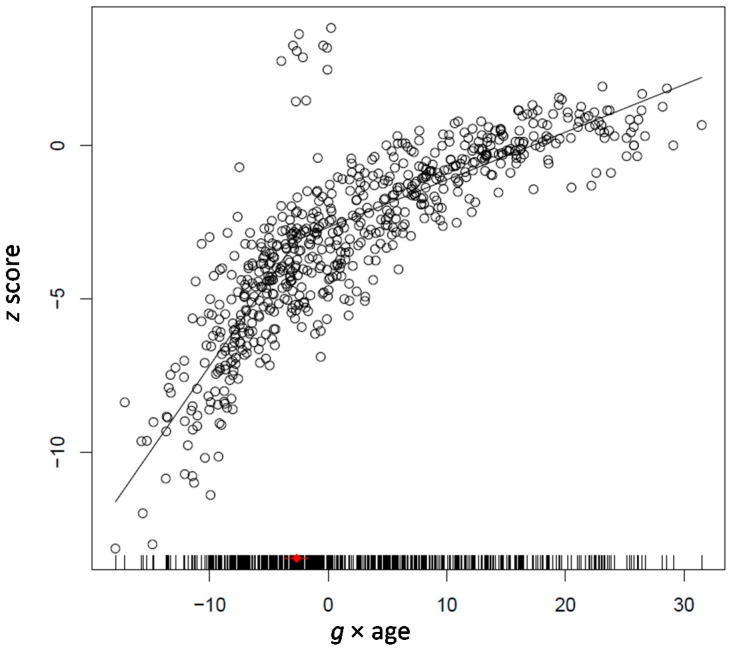
Development of attention control as a function of developmental *g* (*g* × age) from 4 to 16 years of age. Note: In this and all other figures generated by segmented modeling the developmental *g* (*g* × age) index is used because of the developmental information it conveys. The specific values appearing in each figure reflect the age period involved in the model concerned. Note: In real age, the estimated break point in rate of change (−2.65 points of the *g* × age product) corresponds to ~8 years of age. Statistics for the segmented model: F(2,634) = 108.38, *p* < 0.001.

Figure 3 shows the segmented regression model for attention control as fit on the whole sample of participants from 4 to 17 years of age. It can be seen that change in attention control accelerates with developmental *g* from 4 to 8 years; change slows afterwards, with variation increasing at in the transition point and later on. It is noted that attention control was selected as exemplary of the slopes of all speeded reaction variables used in this and other studies presented here.

Two important conclusions are suggested by these findings, one methodological and one theoretical. Specifically, first, there can be no accurate psychometric modeling of the relation between mental processes independent of developmental phase. At best, modeling of large samples involving individuals widely varying in age range shows a crude image of relations between processes. At worse, this image is useless when one zooms in on specific age phases because the relations vary with phase. The ideal period of time must span over two consecutive developmental phases residing either across two cycles or covering a full cycle. Two consecutive phases expand over enough time to allow possible differentiation in the relations between processes to show up, if present. Thus, comparing these relations across cycles might show how developmental priorities change with growth. Comparing relations within a cycle would show how the various processes of interest are interwoven to produce more complex systems of ability. The studies below focus on each phase.

## 6. The Studies: Modeling Re-Morphing *g*

### 6.1. Study 2: From Realistic Representations to Rules

*Becoming aware*. The present study was initially designed to examine the possible impact of learning the Chinese logographic system on various aspects of intellectual development in the early phases of learning to read. Thus, this study involved children from Greece (*N* = 140) and China (*N* = 159) about equally drawn from each of the age years 4 through 7. The interested reader is referred to the original presentation of this study [12]. For the present purposes, the two ethnic groups were combined to increase power in the relations of interest. In fact, testing the models separately in each ethnic group suggested no ethnic differences in the various factor relations that are of interest here.

These children were examined by a large array of tasks addressed to processing efficiency, executive control, working memory, reasoning, and cognizance. Specifically, for speed of processing, several Simon-like tasks required to specify if simple geometrical figures appeared to the left or the right side of the screen; also children were asked to judge if two letters (Latin, Arabic, or Chinese ideograms) were similar or different. Executive control examined inhibition and shifting. These tasks included a learning phase and a control phase. In the learning phase, children were first trained to touch as fast as possible a picture matching a figure shown screen. In the control phase children were instructed to choose the key *not* showing the figure on the screen. Therefore, the test examines the ability of the child to inhibit a dominant response in favour of the weaker but relevant response “shift to the other one”. Working memory was examined by the Corsi task addressed to spatial working memory and a phonological task involving regular words and pseudowords.

The following types of reasoning were examined: Simple arithmetic reasoning (counting from 3–9 objects and finding the sum of 1 + 2, 2 + 3, and 7 + 4); spatial reasoning (picture assembly and mental rotation, e.g., assemble a square, a triangle and a circle into a house); deductive (pragmatic) reasoning (modus ponens, conjunction, and disjunction; e.g., point to the picture standing for the expression “If Sally wants to play outside, she must put her coat on”) and analogical reasoning (i.e., numerical, spatial, and verbal analogies). These tasks addressed reasoning abilities spanning from the second realistic representations phase to the second phase of rule-based thought. Scoring was based on both the answer given (correct vs. wrong) and the explanations provided for the answer chosen. 

Cognizance was examined by tasks addressed to awareness of mental processes and awareness of the mental demand of tasks. Six of the cognitive tasks summarized above (two from each domain, clearly differing in difficulty) were depicted in separate pictures. For example, for quantitative thought, there was (1) a child adding three cubes and (2) a child adding five cubes. For deductive reasoning, there was (3) a child hearing a story asking her to obey one rule and (4) a child hearing a story asking her to obey two rules. For spatial thought, there was (5) a child reproducing a figure consisting of three components and (6) a child reproducing a figure consisting of five components. Six pairs of pictures were presented to the children: (i) The two addition tasks, (pictures 1 and 2); (ii) the two story-hearing tasks (pictures 3 and 4); (iii) the two figure-reproduction tasks (pictures 5 and 6); (iv) the easy addition and the easy story-hearing tasks (pictures 1 and 3); (v) the easy addition and the easy figure-reproduction tasks (pictures 1 and 5); and (vi) the easy story-hearing and the easy figure-reproduction tasks (pictures 3 and 5). Children were first asked to describe each picture in order to focus on the activities concerned. They were then asked to reflect on (i) the similarity (i.e., “Is the job of *this* child the same as the job of *this* child? Why do you think so?”), and (ii) the relative difficulty of mental processes activated (“Who of the two children is doing the *easier* job? Why do you think so?”) 

To engage the participants in reflection about the mental activities of the children depicted in the pictures, participants were first asked to describe each picture. They were then asked to judge if the two children employed the same thinking and indicate the child having the easier job. Thus, twelve scores (six similarity estimations and six difficulty estimations) were obtained. The first three pairs addressed comparison of tasks belonging to the same domain (quantitative, deductive, and spatial reasoning, respectively) and the rest addressed comparison of tasks belonging to different domains (quantitative-deductive, quantitative-spatial, and deductive-spatial, respectively). Scoring was based on both the answers given and their explanations. Increasing scores reflected a shift of awareness from superficial perceptual characteristics of the tasks (e.g., “they are the same kind of cubes in the two pictures”) attained by late realistic representations to mental processes (e.g., “they are both counting”; “one is counting, the other is classifying”; “it is easier to count few than many cubes”; “it is easier to count than to understand a story”), attained by late rule-based thought.

The Tucker-Drob model was applied on the following mean *z* scores: speed, control, letter recognition, working memory, deductive reasoning, analogical reasoning, cognizance of similarities and cognizance of differences. Notably, the model involving the relations between each process and (i) age, (ii) *g*, and (iii) the age differentiation factor (*g* × age) fit better (AIC = 4903.676) than all three other models (age and *g* = 5019.46; age, *g*, and quadratic *g* = 5149.67; age, *g*, quadratic *g*, and *g* × age = 5156.62). Also, this model fit better than the constrained model where the relation between each process and *g* was constrained to be equal with its corresponding relation with quadratic *g* and *g* × age (AIC = 5473.46) or the constrained model where only quadratic *g* and *g* × age for each process were constrained to be equal (AIC = 4939.92). Obviously, in this age period, the age differentiation factor was enough to capture changes in the formation of *g*. Moreover, the lower fit of the constrained models suggested that these changes varied across processes. 

**Figure 4:**
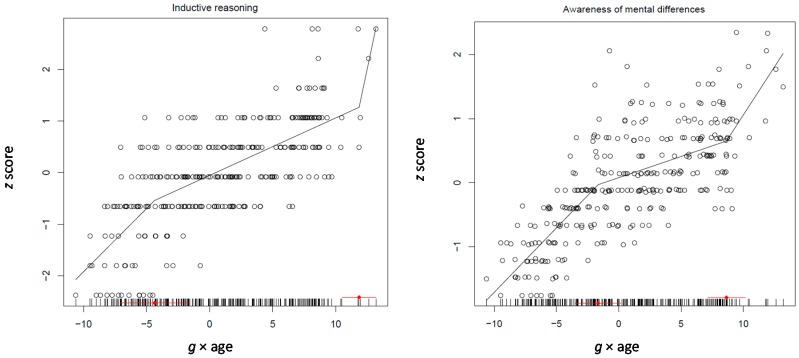
Development of inductive reasoning and awareness of mental differences between processes from 4 to 11 years as a function of developmental *g* (*g* × age). Note 1: In real age, the estimated break point in rate of change in inductive reasoning (−4.8 and 11 points of the *g* × age product) corresponds to ~4 and 9 years of age. Statistics for the segmented model: F(4,293) = 6.68, *p* < 0.001. Note 2: In real age, the estimated break points in rate of change in awareness (−1.65 and 8.65 points of the *g* × age product) corresponds to ~5 and ~7.5 years of age. Statistics for the segmented model: F(4,293) = 7.66, *p* < 0.001.

Only the best fitting model is discussed here. Provided that this study gears on the transition from realistic representations to rule-based thought, one would expect strengthening of relations between inductive reasoning and the age differentiation factor *g* but also between awareness and this factor. This is precisely what is found. The relation between analogical reasoning and *g* × age (0.18, *p* < 0.0001) was significant and positive. The relations of both cognizance of similarities (0.10, *p* < 0.005) and cognizance of differences (0.14, *p* < 0.0001) with the *g* × age were also significant and positive. Notably, the relation between working memory and *g* × age (0.13, *p* < 0.0001) was in the same direction. Interestingly, and in line with the findings of the study above for the 4–6 years old children, the relations of all three speeded performance scores with the age differentiation factor were significant and negative (−0.18, −0.26, and −0.24 for speed, control and reading, respectively; all *p* < 0.0001) (see Table 2; correlations between the variables used in the various models are shown in Supplementary Table S2). These patterns strongly suggest that inductive reasoning and working memory, together with cognizance of inferential processes, build up during the years of transition from representational to rule-based thought. Figure 4 illustrates the pattern of development of inductive reasoning and awareness of mental differences between processes as suggested by segmented modeling.

### 6.2. Study 3: Building Rule-Based Thought

*Becoming aware.* A second study focused on the role of cognizance in the construction of inductive inference that is central in the development of rule-based thought (see [14,16], for details). This study involved 343 children about equally drawn from each of the age years 4 through 11. Specifically, to address processing efficiency, this study involved the Simon-like processing speed tasks, Stroop-like attention control tasks summarized above, and also conceptual control tasks. In these tasks children saw two objects, one bigger than the other side-by-side. The object which was bigger on screen (e.g., a tree leaf) was smaller than the other in reality (e.g., a tree). Their task was to choose the object which was bigger in reality. Working memory measures were similar to those used in the study above. 

Reasoning was addressed by a Raven-like test developed for the present purposes. Matrices addressed three levels of complexity, known to be mastered at 5–6, 7–8, and 9–11 years of age, respectively, i.e., at the phase of late realistic representations, first, and second phase rule-based thought, respectively. Matrices at the first level examined the ability to uncover the pattern defining a single dimension (same color-same size, increasing size, same size-alternating color). Matrices at the second level examined the ability to conceive of the intersection between two dimensions (e.g., animal and color, animal and size, color and size). Matrices at the third level examined the ability to conceive of the intersection between three dimensions (color, shape and size, animal, color, and size, and activity, color, and size) (see [14,16], for details).

Several tasks addressed awareness of perception, associated with realistic representations and inference as sources of knowledge, associated with rule-based thought. In the perceptual awareness tasks children saw a figure placing objects in same color boxes according to their color and heard the figure describing what she did before. Children were then asked to specify the location of objects based on what they saw and heard before. In the inferential awareness tasks, children saw the same figure hiding objects in same color boxes but they were subsequently asked to locate objects of a different color not shown before. Thus, this condition addressed awareness of inductive extrapolation as a source of knowledge. That is, that this not-seen before object must be in a same-color box, given that the figure is placing objects in same color boxes.

The model was first tested on the whole sample of 343 children (see Table 3; correlations between the variables used in the various models are shown in Supplementary Table S3). This model involved a measure for speed, attention control, conceptual control, working memory, a score for each of the three Raven levels, a score for perceptual awareness, and a score for inferential awareness. In this model, all three speeded performance measures differentiated significantly from quadratic *g* (−0.22, −0.16, and −0.16 for speed, attention control, and conceptual control, respectively, all *p* < 0.01). Of the various Raven and working memory scores, one (Raven B, −0.16, *p* < 0.05) also differentiated from quadratic *g*. However, the two awareness measures related positively and significantly with quadratic *g* (0.13, *p* < 0.05, and 0.31 *p* < 0.001, for perceptual and inferential awareness, respectively). Notably, perceptual awareness was negatively related to the *g* × age product (−0.16). Therefore, this model suggested clearly that awareness of mental processes is a crucial factor in the formation of *g* at the end of the representational cycle and practically all of the cycle of rule-based thought.

To zoom in on these processes the model was applied separately on the 4–6 (*N* = 172; AIC = 5852.28) and 7–11 years old children (*N* = 171; AIC = 5656.00). It is noted that in both cases, the full unconstrained model fit better than any of the other models omitting any of the factors (all AIC > 5862.03 and 5691.82 for the two groups, respectively) or holding them equal (all AIC > 5884.78 and 5666.86 for the two groups), respectively). The differences between the two groups are very informative for the dynamics of *g* formation. Specifically, of the various processes, only perceptual awareness was significantly and positively related with quadratic *g* in the 4–6 years old group (0.27, *p* < 0.0001). In the 7–11 years old group both, perceptual (0.15, *p* < 0.0001) and inferential (0.18, *p* < 0.007) awareness were positively related to quadratic g. Additionally, in this group, both speed (0.10, *p* < 0.02) and inhibition (0.12, *p* < 0.007) were positively related to quadratic *g*. Notably, Raven A (−0.57, *p* < 0.0001) and Raven C (−0.15, *p* < 0.009) were negatively related to quadratic *g*. Therefore, these patterns suggest that perceptual awareness builds up in the representational cycle. This continues to get intertwined with increases in *g* in the next cycle of rule-based thought. However, additionally, in this cycle, inferential awareness emerges as a component of *g* development. Interestingly, this goes, on the one hand, with changes in speed and conceptual control, indicating enhanced control of these processes. On the other hand, with increasing g, performance on the Raven matrices starts to vary.

### 6.3. Study 4: Becoming Logical

The studies above showed that the grasp of inferential awareness is an important part of the transition from representational to rule-based thought. The present study focused systematically on the development of inductive and deductive reasoning from ~7 to ~12 years of age. This study involved children (*N* = 395) about equally drawn from each of the six primary school grades. Mean age was 6.7, 7.9, 8.9, 9.8, 10.7, and 11.7 years, respectively. A large number of tasks addressed to various aspects of speed and control of processing, working memory, and deductive and inductive reasoning were used (see [25,43] for details). 

*Speed and control of processing*. The Simon-like task described above addressed speed of processing. A series of Stroop-like incompatible tasks addressed perceptual control. The tasks addressed to conceptual control described above were used. 

*Visuo-spatial working memory*. To test visuo-spatial memory, several arrangements of geometrical figures of varying complexity (2–5 figures) were presented to the participants. The participants’ task was to choose one among four alternative arrangements matching the arrangement presented before. To test *numerical working memory*, a set of number digits, differently colored, were presented in succession (2–7 digits). At the end of the presentation of each set, a target digit was presented and the participant’s task was to specify if this target digit was bigger than the same color digit included in the set. Four trials were given for each level of difficulty. Participants had to succeed in at least two of the four trials in order to move on to the next level. In a second task, the numbers were represented by dots of equal size rather than by number digits. Participants had to keep in memory both the numerical information and the color of the items presented in each trial to succeed. Thus, this task was more demanding in that both numerical and color information would have to be stored and recalled [26]. 

*Reasoning.* A long battery addressed inductive and deductive reasoning through tasks involving verbal, mathematical, and spatial relations. Specifically, there were 12 verbal *inductive reasoning* tasks, five syllogisms and seven verbal analogies. The syllogisms required one to make an induction about a particular case in a story based on the characteristics of a group of cases. In the verbal analogies one of the four components was missing and participants chose the right answer among four alternatives (i.e., a:b::c:?). In the same fashion, number syllogisms asked participants to make inductions about relations between numbers. In the mathematical analogies, the children chose the missing number of a pair based on the relation between a complete pair (e.g., 3:4::6:?). Spatial syllogisms addressed the ability to extract a general rule underlying movement in a matrix of varying complexity according to several rules. Raven-like matrices were addressed visuospatial analogical thought. 

*Deductive reasoning* was addressed by verbal, mathematical, and spatial reasoning tasks. Sixteen standard arguments addressed verbal propositional reasoning. These arguments involved two premises and a conclusion and the participant’s task was to indicate whether the conclusion was right, wrong, or undecidable. Arguments addressed: modus ponens, modus tollens, the fallacy of affirming the consequent, and the fallacy of denying the antecedent. *Mathematical syllogisms* addressed the same relations in numerical context. *Spatial syllogisms* asked participants to deduce the position of an animal or a person based on the information of a number of propositions constraining each other in the fashion of the mathematical reasoning tasks described above. 

These reasoning tasks addressed three developmental levels. At the first level of inductive reasoning, children identify patterns and formulate generalizations on the basis of a single dimension or relation. At the second level, they can handle *hidden* or *implied* relations that require from the thinker to combine information present to the senses with knowledge stored in long-term memory. Finally, at the third level, multiple parameters and relations would have to be simultaneously considered and manipulated. At the first level of deductive reasoning modus ponens inferences can be handled, simple at the beginning (involving only affirmative premises) and more complex latter on (negations may be involved). At the second level, modus ponens is integrated with modus tollens, indicating the ability to construct models which take the modus ponens argument as a basis and then construct alternative models which are compared to each other. Finally, at the third level of deductive reasoning the fallacies can be solved. The thinker at this level must accept that not all arguments are determinate and thus uncertainty may be part of the reasoning process itself. These tasks span early rule-based thought through late principle-based thought.

**Figure 5:**
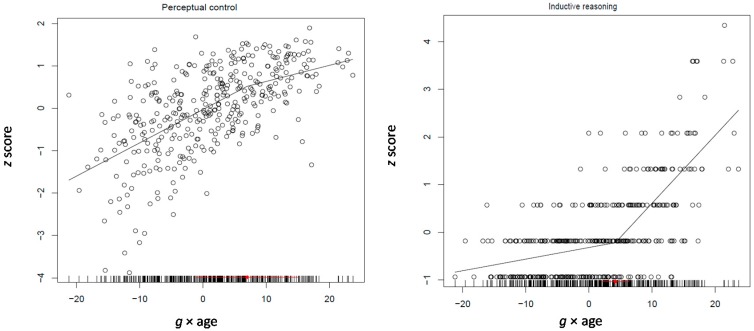
Perceptual control and Level 3 inductive reasoning as a function of developmental *g* (*g* × age) in the rule-based cycle (7–12 years of age). Note 1: In real age, the estimated break points in rate of change (6.91 points of the *g* × age product) corresponds to ~9 years of age, but it was not significant, F(2,371) = 2.31, *p* > 0.05. Note 2: In real age, the estimated break point in rate of change (4.19 of the *g* × age product) corresponds to ~8.5 years of age. Statistics for the segmented model: F(2,371) = 31.47, *p* < 0.001.

For the present purposes, three processing efficiency (speed, attention control, conceptual control), three working memory (visuospatial, numerical, integrated numerical-symbolic), and six reasoning scores (three inductive and three for the deductive reasoning levels) were used in the model. The model involving all four factors (age, *g*, quadratic *g* and *g* × age) fit better than the models where any of the two differentiation factors were omitted (all AIC > 11,180.58). Notably, however, the model where quadratic *g* and the *g* × age factor were constrained to be equal fit better (AIC = 11,128.37) than the unconstrained model (AIC = 11180.58). The results were clear. It can be seen that of the various relations, level 3 of inductive reasoning relates positively and significantly with both quadratic *g* and the *g* × age product (0.20, *p* < 0.0001) (see Table 4 and Figure 5; attention control is also illustrated for indicative purposes; correlations between the variables used in the various models are shown in Supplementary Table S4). Interestingly, of the three deductive reasoning levels, level 2 of deductive reasoning related weakly but significantly to both differentiation factors (0.05, *p* < 0.04). Thus, in the age period from 7 through 12 years, advanced inductive and solid but less than optimum deductive reasoning get integrated into *g* together with increased inferential awareness suggested by the studies presented above. It is notable that testing the model separately on 7–9 and 10–12 year old children yielded practically identical results.

### 6.4. Study 5: Consolidating Principle-Based Thought

Several studies focused on the development of principle-based thought. The first of the studies to be presented here focused on the transition from rule-based to principle-based thought, the consolidation of the latter, and the role of various factors of processing efficiency in this dynamics (see [44], for details). This study involved 478 participants, about equally drawn among 6 through 17-year olds. For the present purposes, only the 11–17 year olds participants were involved (*N* = 289). These participants were examined by tasks addressed to processing efficiency, working memory, mathematical reasoning, and fluid intelligence. Specifically, processing efficiency was addressed by Simon-like processing speed tasks, Stroop-like attention control tasks, and several divided and selective attention tasks. Working memory was addressed by the forward and backward digit span tasks included in the WISC-III test. 

Mathematical reasoning was addressed by tasks examining the ability to execute arithmetic operations in combination to each other, algebraic reasoning, and proportional reasoning. Items in each domain scaled along four levels. In the arithmetic tasks, participants were asked to specify the operations missing from simple arithmetic equations: One (e.g., 5 * 3 = 8), two (e.g., {4 # 2} * 2 = 6), three (e.g., {3 * 2 # 4} @ 5 = 7), and four operations (e.g., {5 @ 2} o 4 = {12 $ 1} * 2) were missing from the items of each level. The algebraic reasoning tasks required to specify one or more unknowns in an equation (e.g., *a* + 5 = 8, specify *a*; *u* = *f* + 3; *f* = 1; specify *u*; if (*r* = *s* + *t*) and (*r* + *s* + *t* = 30), specify *r*; when is true that {*L* + *M* + *N*} = {*L* + *P* + *N*}? for the four levels, respectively). In proportional reasoning, the four levels required to grasp relations between the following: (i) fully symmetrical and equivalent ratios (e.g., ½ to 3/6); (ii) equivalent but not obviously symmetrical ratios (e.g., 2/6 to 3/9); (iii) ordered pairs with two corresponding terms multiple of one another (e.g., 2/5 to 3/7); (iv) pairs without corresponding terms (e.g., 5/12 to 3/8). In terms of the cycles of development specified in the introduction, the two lower levels of these batteries are primarily related to the two phases of the rule-based concepts. Levels three and four addressed the two phases of the principles cycle, respectively. 

Raven’s Standard Progressive Matrices involve five sets of matrices of increasing complexity. Based on Rasch scaling of performance on each of the 60 matrices, four levels were formed, each involving 15 matrices. From easy to difficult, matrices in the first group, require grasping the pattern underlying figures varying along a single dimension. In the second group, two familiar and obvious dimensions (e.g., shape, size, background, etc.) would have to be integrated. In the third group, matrices require deciphering and integrating critical dimensions through systematic search and transformation of the features involved. For instance, it is the double of …, it goes by one more, etc. Finally, in the fourth group, matrices require deciphering multiple dimensions by grasping the thread underlying several transformations of figures and integrating into complementary general principles. Level 1 addresses abilities of the second phase of the representational cycle. Levels 2 and 3 address abilities associated with the two phases of rule-based thought, respectively. These were the levels represented in the Raven-Like test used in the study described above. Level 4 addresses abilities of first level of principle-based thought.

**Figure 6:**
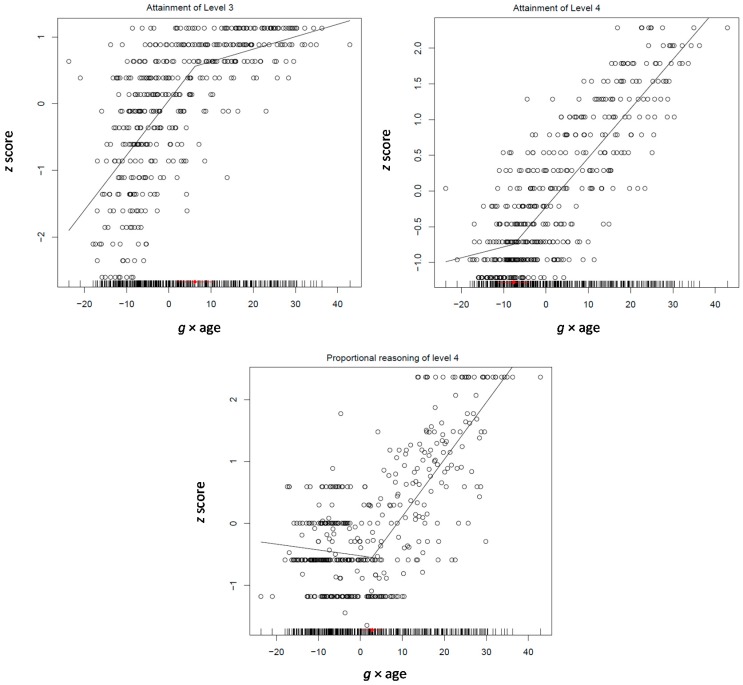
Attainment of Level 3 and Level 4 in Raven’s Standard Progressive Matrices as a function of developmental *g* (*g* × age) from 11 to 17 years of age. Note 1: In real age, the estimated break point for Raven Level 3 (6.20 of the *g* × age product) corresponds to ~11 years of age. Statistics for the segmented model: F(2,426) = 17.84, *p* < 0.001. The estimated break point for Raven Level 4 (−7.60 of the *g* × age product) corresponds to ~9 years of age. Statistics for the segmented model: F(2,426) = 7.25, *p* < 0.001. The estimated break point for Proportional Reasoning Level 4 (2.82 of the *g* × age product) corresponds to ~10.5 years of age. Statistics for the segmented model: F(2,426) = 51.63, *p* < 0.001.

The model was first applied on three measures of processing efficiency (speed, attention control, and divided attention), working memory, four mean scores standing for performance on the four levels of mathematical reasoning across the three domains (i.e., arithmetic, algebraic, and proportional reasoning) and the four levels of Raven’s Standard Progressive Matrices. The model involving only three of the factors (age, *g*, and quadratic *g*) fit better (AIC = 4954.93) than the models involving only the first two (AIC = 5179.49), *g* × age instead of quadratic *g* (AIC = 5065.32), or all four factors (AIC = 5093.00). In fact, the model where quadratic *g* and the *g* × age factor were constrained to be equal fit better (AIC = 4937.93); dropping the relation between Raven level 4 and the *g* × age factor resulted in a slight improvement of the model fit (AIC = 4933.10). This is an interesting model (see Table 5; correlations between the variables used in the various models are shown in Supplementary Table S5). Mean mathematical level 3 (0.02, *p* < 0.05) and 4 (0.03, *p* < 0.001) was positively, albeit weakly but significantly, related to both quadratic *g* and *g* × age. The relation of Raven level 4 with quadratic *g* was much stronger (0.09, *p* < 0.0001). Raven levels 1–3 related negatively with both differentiation factors (−0.06–−0.09, all *p* < 0.001) (see Figure 6, illustrating attainment of Level 1 and Level 4). It is clear that the two higher mathematical thought levels and the fourth Raven level infuse *g* in the period from 12 to 17 years, suggesting that in the phase of principle-based thought the major acquisition of *g* is strategic abstraction and formation into abstract relations. With increasing ability, variation of performance on lower levels increases.

It would be interesting to zoom in on each of the three aspects of mathematical reasoning because, despite their overlap, each stands for specific mental processes not captured by the other two. Arithmetic reasoning expresses the basics of mathematical reasoning. Algebraic reasoning captures deductive logical relations running through mathematical objects. Quantitative proportional reasoning as measured here captures relational thought integrated into mathematical reasoning and related computational skills that enable the thinker to transform a relation between relations into general principles and mathematically specify them. To specify possible differences in the contribution of each of these processes to the formation of g, the model was separately tested on each process. Interestingly, the relations of both arithmetic and algebraic reasoning with both quadratic *g* and the *g* × age product, if significant, were always negative. Therefore, increasing ability goes with increasing variation of these two processes. In contrast, however, levels 2–4 of proportional reasoning were positively and significantly related to both quadratic *g* (0.15, 0.18, 0.21, for levels 2–4, respectively, all *p* < 0.0001). It is strongly suggested that the major acquisition of *g* in the principle-based cycle is a formalization process that enables transforming relations into precise mental representations formally stated. 

### 6.5. Study 6: Becoming Meta-Logical and Meta-Cognitive

It is interesting to examine if these formations go with cognizance. To answer this question, we modeled the results of a study that focused on the possible relations between the acquisition of principle-based abilities and cognizance about them in adolescence (see [21], for details). This study involved a total of 621 participants about equally (*N* = ~90) drawn among 7th to 12th secondary school grades (mean 11.5 to 17.4, respectively), university students (29) and secondary school teachers (20). These participants were examined by a pair of tasks addressed to each of four domains: mathematical proportionality, deductive, causal-experimental, and spatial reasoning. The first task of each pair addressed first (symmetrical ratios, modus tollens, isolation of variables, mental rotation of two figures in coordination) and second level principle-based thought (non-symmetrical ratios, matching hypothesis with 2 × 2 experiment, grasping fallacies, and coordination of shadow projections of geometrical figures inversely varying). 

Participants were asked to evaluate their success on each task on a 4-point scale (not, slightly, quite, very satisfied). These scores were then combined with the corresponding performance score on each task to yield the self-evaluation accuracy score for each task. Participants were also asked to judge the procedural-processing similarity of 22 pairs of tasks belonging to various combinations of domain and level affiliation (e.g., tasks addressed to the same domain and level, same domain but different level, different domain and level, etc.). Participants were instructed to rate the similarity of the tasks in each pair “with respect to the ways of thinking you applied when trying to solve them; take into account how your mind worked when solving each task” (not similar, slightly, quite, very similar). They were then asked to explain three of their similarity judgements (pairs involving two causal, two spatial, and one causal and one quantitative task). These similarity judgements were scored on a 4-point scale (irrelevant or wrong responses, similarity based on content task characteristics, global identification of mental processes involved, complete specification of mental similarities and differences). Obviously, accuracy of success evaluation and similarity judgments capture two complementary aspects of cognizance. The first captures a system of standards enabling one to monitor, evaluate, and adjust problem solving accordingly; the second captures explicit awareness of the mental processes involved. Obviously, mental monitoring and regulation would be easier when thinkers possess both self-evaluation standards and explicit awareness of the processes they might have to regulate to improve performance. Under these conditions, thinkers may be able to do both: (i) notice possible deviations between solutions produced and the best solution possible as indicated by the standards; (ii) explicitly select a process that is best conducive for the production of the best possible solution.

The model involved the four domain-specific mean performance scores, the four mean self-evaluation accuracy scores, and the mean similarity evaluation score. The full unconstrained model involving all factors fit better (AIC = 14,387.15) than the unconstrained models where any of the two differentiation factors were omitted (AIC = 14,572.74 and 14,488.14, for quadratic *g* and age differentiation, respectively) or they were held equal to each other and *g* for each process (AIC = 14,870.80) or only to each other (AIC = 14,429.12). The patterns of relations obtained (see Table 6; correlations between the variables used in the various models are shown in Supplementary Table S6) are highly informative: First, performance on both causal and mathematical thought was not related to any of the two differentiation factors; self-evaluation in both was weekly but significantly related to *g* × age (−0.05 for both, *p* < 0.03). Second, both performance and self-evaluation on spatial thought were negatively related to both quadratic *g* (−0.16 and −0.22, *p* < 0.0001, respectively) and *g* × age (−0.10 and −0.10, *p* < 0.0001, respectively). However, third, performance (0.18, *p* < 0.0001) and self-evaluation (0.15, *p* < 0.0001) on deductive reasoning was positively related to quadratic *g*. Notably, awareness of similarity between processes was positively related to both quadratic *g* and *g* × age (0.23 and 0.13, *p* < 0.0001, respectively). It is clear, therefore, that the build-up of *g* during the formation of principle-based thought is based on the grasp of the constraints underlying inference drawing in deductive reasoning, awareness about these constraints, and also awareness of mental processes as such. At the same time, other processes may increase linearly with *g* (e.g., causal and mathematical) or differentiate from it (spatial reasoning).

**Figure 7:**
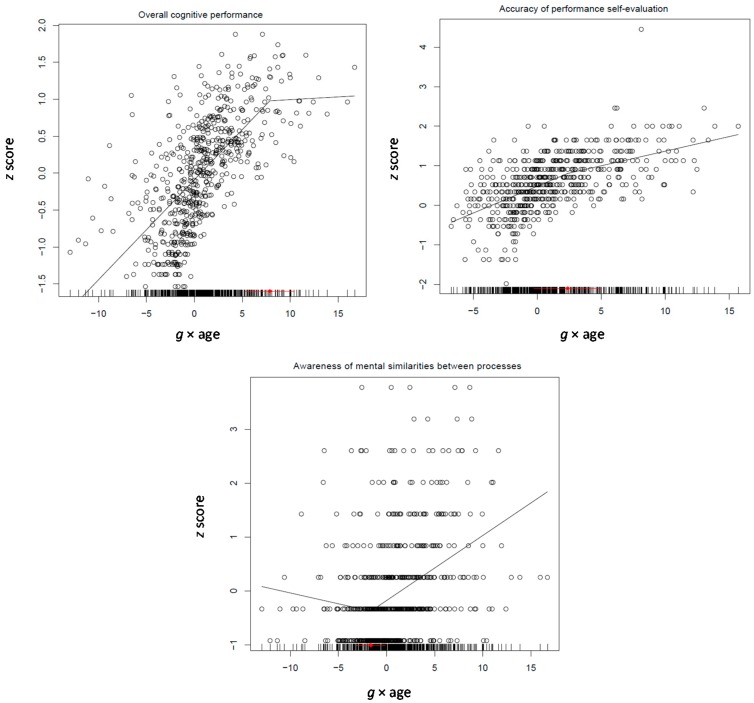
Overall cognitive performance, accuracy of performance self-evaluation, and awareness of mental similarities between processes as a function of developmental *g* from 12 to 17 years of age. Note: The estimated break point for overall cognitive performance (8.78 of the *g* × age product) corresponds to ~16 years of age. Statistics for the segmented model: F(2,617) = 6.42, *p* < 0.001. The estimated break point for self-evaluation (2.38 of the *g* × age product) corresponds to ~14 years of age. Statistics for the segmented model: F(2,617) = 7.61, *p* < 0.01. The estimated break point for awareness of similarities (−1.62 of the *g* × age product) corresponds to ~14.5 years of age. Statistics for the segmented model: F(2,617) = 11.40, *p* < 0.001.

Segmented modeling (see Figure 7) showed that reasoning takes off at the beginning of this period at 11.5 years and levels off at 16, which is expected for the development of principled reasoning. Self-evaluation spurts in the middle of this period around 14 years and awareness of similarities spurts about one year later at 14.5 years. Thus, it seems that incipient grasp of principled thought is intertwined with awareness about it before it is consolidated by the end of this cycle. 

### 6.6. Changes through the Life-Span

Many argued that changes in adulthood gradually follow the inverse course of change as individuals approach old age [45]. There is evidence showing that all mental processes eventually decline with age, although the age of peak and fall differ across processes. For instance, processing efficiency and working memory peak and decline earlier than reasoning and crystallized abilities [46]. Decline was taken to imply de-differentiation of specific processes from *g* with senility. That is, mental processes come to depend more on *g* as they weaken due to impairment in central processes [4,38,47]. We conducted several studies on intellectual development which focused on changes from adolescence through 85 years of age. Two of these studies will be presented here.

### 6.7. Study 7: Processing Efficiency, Working Memory and Inductive Reasoning from 11 to 85 Years

The first of these studies involved 468 participants about equally drawn among 11 to 85 years old participants. These participants were examined by the processing efficiency tasks used in Study 4 (processing speed, perceptual discrimination, perceptual control and conceptual control), two working memory tasks and an inductive reasoning battery that was first used here.

The Inductive Reasoning Developmental Test (IRDT) is a pencil-and-paper battery designed to assess developmental levels spanning from early childhood to adulthood [48]. It is based on the hierarchy of cognitive levels specified in Commons’s Model of Hierarchical Complexity [49]. This model basically integrates Piagetian stages with their neo-Piagetian elaboration and formalizes them in mathematical terms. Commons’s hierarchy involves 16 hierarchical levels, spanning from very early sensorimotor actions to late post-formal reasoning. In Piagetian terms, the present test involved tasks spanning seven levels from Piagetian-like preoperational to post-formal metasystematic reasoning. Obviously, these levels correspond to the phases spanning from the second phase of realistic representations through the second phase of principle-based thought. Post-formal metasystematic reasoning would be considered a kind of advanced principle-based thought requiring the integration of several principles into integrated higher-order hierarchies of principles. This sequence is defined in reference to three mathematical axioms: (i) Each next order of hierarchical complexity (or sub-stage) is defined in terms of tasks at the next lower order; (ii) higher order constructs specify the way in which the less complex actions combine; and (iii) the lower order task actions have to be carried out non-arbitrarily, constrained by higher-order constructs. 

In the present battery, tasks required to specify the rules underlying the relations between letters or sets of letters of the alphabet. There were four sets as distinct cases implementing the relation concerned and a set violating this relation. The task was to discover the exception. Specifically, in the first level (pre-operational), there was a sequence of five letters, four similar and one different: AAA***E***A. At the next level, there were pairs of related letters linked according to a specific rule (e.g., consecutive letters) with a pair violating this rule: WX, KL, ST, ***PR***, YZ. At the next level (concrete), items involved a rule connecting a more complex sequence of letters along two dimensions, such as adjacency and position: NOPR, IJKM, ***UVXY***, MNOQ, QRSU. The next (abstract) level involved sets of four letters arranged in two pairs the one above the other (e.g., $\frac{\mathrm{EF}}{\mathrm{JH}}$). The participant needs to specify the relationship between E and F (no letter between them), F and H (one letter missing in between), H and J (one letter missing in between), as well as between J and E (four letters missing in between). The systems are: E-F-H (system 1), H-J-E (system 2), and they are reversible, so it goes forth and back (from E to J and J to E). There were five such sets, four governed by these relations and one exception. At the next level, (formal) items required the participants to make a logical induction through the analysis of coordinated abstract, general, class of systems. The participants should proceed from the identification of the relation between three coordinated abstract variables: (e.g., $\frac{\mathrm{AB}\;}{\mathrm{JD}}\;\frac{AF\;}{BD}\;\frac{\mathrm{DF}\;}{BA}$). At the next level, (systematic) items required to identify a general principle based on the analysis of pairs of coordinated abstract rules, forming a system of relations within a single option. Four options present a pattern where the first pair of mapped abstract variables have distance 6, e.g., A to H, while the second pair have distance 3, e.g., H to L. For instance: (e.g., $\frac{\mathrm{AB}\;}{\mathrm{FD}}\;\frac{\mathrm{HM}\;}{\mathrm{IK}}\;\frac{\mathrm{LN}\;}{\mathrm{JK}}$). Finally, meta-systematic items required inductions based on the comparison of systems of abstract systems. The first option in the example shown in parenthesis below shows B presenting distance 3 from F; F presenting distance 6 from M and −2 from C. Summing 3, 6 and −2, we have the broad rule of the systems, i.e., 7. All the other options present the same broad rule, except option 3, since E presents distance 2 from Q; Q presents distance 6 from X and −3 from M. Summing these distances we find 5 instead of 7: (e.g., $\frac{\mathrm{BC}\;}{\mathrm{GE}}\;\frac{\mathrm{FH}\;}{\mathrm{DI}}\;\frac{\mathrm{CH}\;}{\mathrm{DF}}\frac{\mathrm{MO}}{\mathrm{KJ}}$). 

For the present purposes, four scores were estimated by Rasch analysis: A comprehensive score standing for performance on the whole battery and three level-specific scores. The first of these scores involved all pre- and concrete level items, addressing rule-based thought; the second involved all formal and systematic items, addressing principle-based thought; the third involved all metasystematic and paradigmatic items, addressing advanced principle-based thought. 

The Tucker-Drob model was applied on two age groups: 11–30 and 31–85 years old participants (see Table 7; correlations between the variables used in the various models are shown in Supplementary Table S7). At a first run, this model involved four measures for processing efficiency, short-term and working memory, and the overall logit score standing for performance on the inductive reasoning test (see Table 7). Interestingly, all four processing efficiency measures differentiated with increasing *g* (−0.10, −0.17, −0.08, and −0.07, all *p* < 0.0001). Working memory was not related to any differentiation measure. Reasoning intertwined with the *g* × age factor (0.12, *p* < 0.02). The pattern was very similar in the 31–85 years age group. All four processing efficiency measures differentiated from increasing *g* (−0.17, −0.26, −0.07, and −0.08, all *p* < 0.05). In addition, working memory also differentiated from *g* (−0.16, *p* < 0.008). However, in this age period, reasoning was not related to any of the two differentiation indexes. Obviously, this pattern of relations does not support a de-differentiation hypothesis as stated above.

To further explore possible differences between age groups in the relations between reasoning and the two differentiation factors, the general reasoning score was substituted by the three level-specific scores. Comparing the two models suggests an interesting difference between the two age groups. On the one hand, 11–30 years is obviously a period of intense reasoning changes gearing on principle-based thought. In this period, principle-based thought intertwined with quadratic *g* (0.22, *p* < 0.008) but also with *g* × age (0.06, *p* < 0.0001). Advanced principle-based thought intertwined, albeit weakly, with *g* × age (0.04, *p* < 0.04). In the 31–85 years period principle-based thought continued to be central as it intertwined with quadratic *g* (0.20, *p* < 0.0001). However, in this period we evidenced a return of rule-based thought (0.17, *p* < 0.003) and a complete disappearance of advanced principle-based thought. We take these patterns to reflect the bending of reasoning power that occurs as age progresses to senility. 

**Figure 8:**
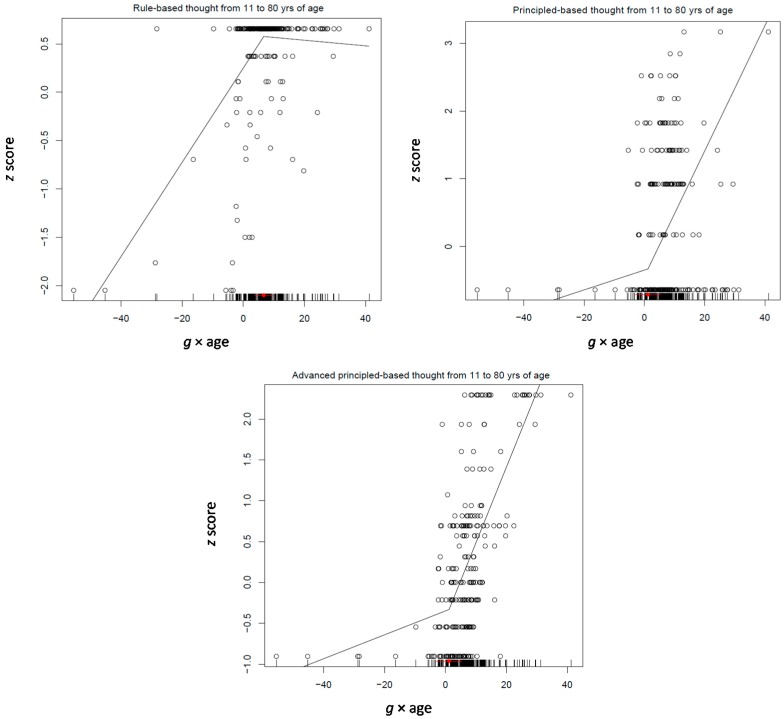
Rule-, (estimated break point = 6.70, age ~22) principled- and advanced principled-based (both estimated break points at 1.2, age ~20) thought age as a function of developmental *g* from 11 to 80 years of age.

These patterns are reflected in the segmented models of change along the age span from 11 to 85 years (see Figure 8). It can be seen that when rule-based (concrete) thought levels off principle-based (formal and post-formal) thought spurt.

### 6.8. Study 8: Reasoning, Self-Evaluation, and Cognitive Self-Representation from 14 to 45 Years

The study above highlighted changes in processing efficiency, working memory and reasoning through the life-span. However, that study did not involve any measures of cognizance. The study to be presented here focused on the relations between reasoning development and self-evaluation of reasoning performance and cognitive self-representation from adolescence to middle age. Thus, it can highlight how these two aspects of self-awareness relate with *g* in a period of relative cognitive stability. One might expect, based on the analysis above, that reasoning abilities may differentiate from *g* in this period. However, self-representation may intertwine with *g*, as information about one’s cognitive performance is integrated into one’s self-concept, probably influencing choices and activities related to preferred problem-solving domains (see [50], for details about this study).

The study involved a total of 282 participants, drawn from four different age groups separated by an average of ten years, namely adolescence (13–15 years of age), early adulthood (23–25 years of age), middle adulthood (33–35 years of age), and mature adulthood (43–45 years of age). Specifically, the youngest age group involved 42 adolescents (22 males, 20 females) drawn from the last year of compulsory education. Each of the three adult groups was equally divided between a sub-group of 40 persons with university education and a sub-group of 40 persons who completed only compulsory education.

*Reasoning*. The reasoning battery addressed three domains, namely spatial, deductive, and social reasoning. There were three tasks for each domain, drawn from a comprehensive test of cognitive development with known developmental and psychometric properties [51]. The tasks addressed to each domain were chosen to represent three clearly different levels of difficulty, modally attained in early and in late adolescence and in early adulthood adolescence, respectively; thus they addressed principle-based thought. The tasks addressed to spatial reasoning examined mental rotation and the ability to integrate mental images. Difficulty in this task was controlled in reference to the complexity of the figures involved and the effect of rotation on the various components. The tasks addressed to deductive reasoning involved modus tollendo ponens and modus tolens arguments. The first required integrating a disjunctive proposition with a simple affirmative proposition, the second problem involved two disjunctive propositions, and the third required integrating a complete syllogism with a statement that defines its truth value. The social reasoning tasks required to resolve social conflicts on the basis of moral criteria or criteria of personal or social interest. The participant’s task was to evaluate the information presented in the story and take a stance related to the conflicting views presented. Familiarity of condition and complexity of the issues involved defined difficulty in these tasks.

*Self-evaluation of performance*. After solving each of the nine tasks, the participants were asked to evaluate their performance in reference to a seven-point scale (from completely wrong, 0, to absolutely correct, 7). These scores were used to create the self-evaluation accuracy index (SEAI) by combining actual performance with self-evaluation. Specifically, accurate self-evaluation was the condition where success on a given task was associated with a score of 6 or 7 on the self-evaluation scale and failure on the task was associated with a score of 0 or 1. Any other combination of actual performance and self-evaluation was considered to be inaccurate or unclear self-evaluation. A score of 0 was ascribed to inaccurate or unclear evaluation and a score of 1 was ascribed to accurate evaluation, under the definition above. Therefore, there were three SEAI scores for each domain of thought, one for each of the three tasks addressed to each of the three domains. For the purposes of the present modeling, the three SEAIs for a domain were summed up and transformed to a *z* score.

*Cognitive self-representation*. A cognitive self-representation inventory was employed that was first used by Demetriou and colleagues [51]. This inventory probes one’s self-representation about general cognitive processes and characteristics, such as learning, memory, and efficiency of processing, and specialized domains of reasoning, including the three domains addressed by the tasks presented above. For the purposes, only self-ratings about the three domains of interest (i.e., spatial, verbal, and social reasoning) and also about representational efficiency (e.g., working memory) were used. The statements addressed to spatial thought referred to visual memory (e.g., “I retain a very clear picture of things”), facility in thinking in images (“I can visualize how things may be arranged in a place”), and spatial orientation (e.g., “I orient myself easily in a new place”). The statements addressed to deductive reasoning referred to the ability to integrate information from different verbal statements (e.g., “I can easily draw the right conclusion from a series of statements”) or the interpretation of evidence (“I can easily draw the conclusion suggested by the evidence I have”). The statements addressed to social thought referred to the facility in understanding other’s thoughts and feelings (e.g., “I understand easily the intentions of others before they express them”; “I am interested in understanding others’ problems”), involvement in issues of social interest (e.g., “I am interested in participating in discussion groups on social issues”), and responsibility towards others and society (e.g., “When I am not sure how to act, I usually think of the consequences of how I act for other people”). Finally, the statements addressed to processing efficiency referred to self-monitoring (e.g., “I can easily monitor my thoughts”) and self-regulation (“I can easily change how I think about a problem”), efficiency in making conclusions (“I can easily make sense of a conversation”).

**Figure 9:**
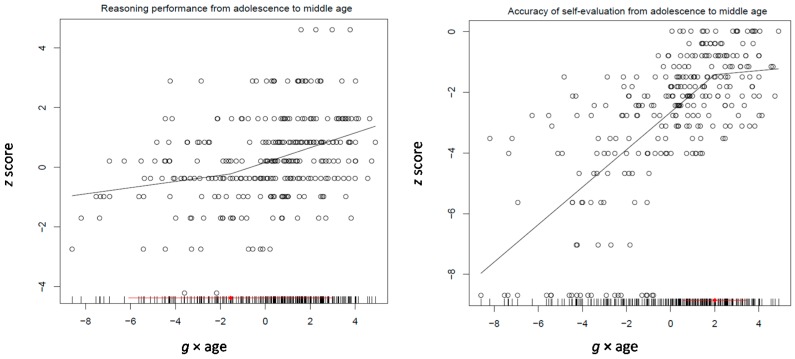
Mean reasoning (estimated break point = −1.55, age ~14, F(2,278) = 0.89, *p* > 0.05) and accuracy of self-evaluation from adolescence to middle age (both estimated break points at 2.01, age ~34, F(2,278) = 3.57, *p* < 0.03)) thought as a function of developmental *g* from 15 to 45 years of age.

The results are summarized in Table 8 (correlations between the variables used in the various models are shown in Supplementary Table S8). It can be seen that there was a clear pattern of differentiation concerning most of reasoning domains in both actual performance and self-evaluation. Specifically, performance in spatial (−0.51, *p* < 0.0001) and deductive reasoning (−0.24, *p* < 0.0001) differentiated clearly from quadratic *g*. In the same direction, self-evaluation in all reasoning domains also differentiated from quadratic *g* (−0.72, −0.31, and −0.18, all *p* < 0.0001, for self-evaluation in spatial, deductive, and social reasoning, respectively). In line with expectations, however, self-representation in deductive reasoning (0.23, *p* < 0.008) and processing efficiency (0.22, *p* < 0.03) did intertwine significantly with quadratic *g*. Segmented modeling (see Figure 9) showed that reasoning spurts in adolescence but cognizance develops throughout early adulthood, peaking at about 34 years of age. 

## 7. Discussion: Realities of the Mind

*Re-morphing g: An integrated/differentiated construct*. Let us first summarize the structural and developmental implications of the eight studies presented here. First, all studies suggested that *g* is a multidimensional psychological construct. Altogether, *g* is a focus (executive control), search and align (flexibility), cognize and choose (awareness and regulation), and inference (deductive and inductive reasoning) and abstract (metarepresentation) mechanism. Each of these processes is autonomous and distinct contributor to *g* at any age. 

Second, all of these processes are always present in development. However, their relative contribution varies. Overall, the contribution of attention control and flexibility diminishes but the contribution of working memory, cognizance, and inference increases with age. This occurs in cycles where the relations between processes are re-worked anew in each cycle. The relations between specific processes and *g* which are integrated into *g* in each cycle are strengthened to reflect that *g* gradually expands to absorb these processes. The major *g* acquisition in the representational cycle is attentional control and flexibility (culminating 4–6 years). Awareness of perceptual processes and the perceptual origins of knowledge are the major markers of *g* expansion. In the rule-based cycle, inductive inference and awareness of it are the major markers of *g* expansion (culminating at 8–10 years). Finally, in the cycle of principle-based thought, grasp of logical constraints framing inference, explicit awareness of mental processes, and criteria for inference are the major markers of *g* (culminating at 14–16 years). In adulthood, these processes strengthen up to middle age. In fact, cognitive self-representation becomes a powerful component of the formation *g*. Later, as people grow towards old age, advanced principle-based thought seems to yield to rule-based thought. 

Third, the changes in the relations between the ever present constitutional processes of *g* transform representational and inferential possibilities with growth making thought to appear to an observer as qualitatively different. Mental units differ at successive developmental cycles (action episodes, words and mental images, rules, principles). However, these mental units that appear qualitatively different can all be defined by varying combinations of the same quantitative parameters. In line with the present findings, we showed earlier that the relations between the development of reasoning, speed, and working memory recycle such that at the beginning of cycles reasoning related with speed and attention control more than with working memory and at the end of cycles this relation weakened while the relation with working memory strengthened [15,25]. The present findings highlight the mechanism of this recycling. The strong relations between *g* and processing efficiency at the beginning of cycles reflect formation of the new cycle-specific mental units where automation in their command is of primary importance and dominates. The high *g*–WM relations at the end of cycles reflect awareness processes suggesting reconstruction of relations between processes that will generate the new mental constructs which will open the way for transition to the next cycle.

These patterns suggest a clear answer to the question that motivated this article. Specifically, differentiation and convergence of specific abilities and *g* are two aspects of the same reality. One of the aspects is a powerful developmental process underlying the formation of *g*. With development in each cycle, the various specific abilities get increasingly intertwined with increasing *g*, according to the representational and inferential needs of the phase concerned. However, there is an equally powerful individual differences process. Relatively well established abilities are increasingly differentiated from *g* in each cycle. This emerges powerfully in the years of mental stability, from early youth to middle age. This simply reflects that with increasing *g* or age in a cycle, variation between individuals may increase to reflect the fact that the range of performance is higher among higher ability individuals, because these individuals have more options and fewer constraints in investing their ability in different realms. It is notable that the pattern of differentiation from and intertwining of processes with *g* conforms to the general model of non-linear growth bringing differential and developmental theory together under a common meta-theoretical scheme. Notably, Reynolds [32] recently showed, in line with the present patterns, that variability and non-linear relations between *g* and broad abilities increases with increasing *g*. 

One might object here that the re-morphing of *g* captured by our modeling methods reflect differences in the tasks batteries used in the various studies rather than differences in the true composition of *g* across developmental cycles. Although accurate to some extent about battery differences across studies, the objection is not tenable for two reasons. First, all studies involved tasks addressed to the same modality and requiring the same type of response. This is the case for all attention and executive control tasks and the working memory tasks used in all studies. Even the reasoning and awareness tasks which may have differed across studies, especially when addressed to participants of distant ages, were designed to vary along the same conceptual and complexity dimensions. In fact, phase-overlap between studies addressed to adjacent developmental cycles yields confidence to the assumption that the *g* processes captured across studies varied along a common continuum. 

It is a truism in developmental science that cross-sectional studies may confound developmental processes with educational, social, and cultural influences. In the present studies, this would imply, for instance, that changes in integration/differentiation patterns in *g*-specific ability relations reflect differences in school learning experiences associated with different age periods rather than genuine developmental processes. The studies presented here do not rule out this possibility because they were all cross-sectional. Therefore, longitudinal validation of changes in these patterns is needed before they are accepted as genuine developmental phenomena. 

*Relations with other theories*. An epistemologist would see several psychological realities in the patterns abstracted by the approach adopted here. We will name these realities after great psychologists or philosophers to show how the theory presented here relates to and builds on earlier theories. 

First, there is definitely a *Piagetian reality*. There are four developmental cycles with two phases in each that for many would be quite close to the four Piagetian stages of cognitive development (sensori-motor, preoperational, concrete and formal operational intelligence). These cycles are representationally and procedurally rather than logically defined. That is, they are distinguished from each other in reference to the type of representation dominating in each cycle (i.e., episodic schemes, mentations, rules, and principles) and by the relations connecting representations (i.e., spatially and time-based associations, representational mappings, inferential links, truth- or validity-based inferential constraints). However, although overlapping in time, they follow a necessary sequence and each next cycle integrates all earlier ones. Piagetian reality is associated with a Gödelian reality. That is, each current phase acquires its full potential in the next phase and each cycle comes to a closure only when moving into the first phase of the next cycle.

There is also a *Spearmanian reality*. The level of general mental ability available at a given time constrains specific mental processes. Consolidated processes may differentiate and vary rather freely in more able (often older) persons. However, change in *g* is by definition developmentally constrained. Individual differences in this regard are actually differences in rate of attainment and infusion of *g* by processes specific to a particular phase. To see this ability differentiation, one needs to look at the proper developmental window with developmentally sensitive task batteries. 

Finally, there is also a *Kantian reality*. “The highest principle of Kant’s theoretical philosophy is that all cognition must ‘be combined in one single self-consciousness’” [52]. We showed here that there is a powerful cognizance mechanism that generates and transforms self-awareness of cognitive processes throughout the cycles above. To a large extent, this awareness defines the subjective aspect of mental functioning, raising it from simple computation to representation where information and mental functioning is subjectively meaningful. 

## Figures and Tables

**Table 1:** Differentiation models based on the whole integrated sample (*N* = 662) and according to age.

Ability	Age	*g*	*g*^2^	*g* × age
Integrated sample (*N* = 662; Free parameters = 30; H0 Loglikelihood = −5892.46; Scaling correction factor for MLR = 1.36; AIC = 11,844.92)				
Speed	0.83 **	−2.25 **	−0.21 **	0.36 **
Control	0.63 **	−1.71 **	−0.01	0.32 **
Verbal WM	0.40 **	−0.39 **	−0.20 **	0.01
Visual WM	0.21 **	0.15	−0.05	−0.04
Gf	0.44 **	−0.07	−0.10 *	−0.04
4–6 years (*N* = 112; Free parameters = 30; H0 Loglikelihood = −993.33; Scaling correction factor for MLR = 1.25; AIC = 2046.66)				
Speed	−1.95 **	12.84 **	−3.19 **	3.04 **
Control	−2.24 **	13.15 **	−3.10 **	3.00 **
Verbal WM	0.10	−0.34	0.09	−0.09
Visual WM	0.45 **	1.59 **	0.04	0.30 **
Gf	0.71 **	0.22	−0.04	0.00
7–11 years (*N* = 300; Free parameters = 29; H0 Loglikelihood = −2670.01; Scaling correction factor for MLR = 1.10; AIC = 5398.01)				
Speed	1.10 **	1.21 **	−1.16 **	0.84 **
Control	0.86 **	0.85 **	−0.89 **	−0.26 **
Verbal WM	0.61 **	1.57 **	−0.09	0.49 **
Visual WM	0.35 **	0.08	0.22 **	--
Gf	0.55 **	0.31 **	−0.03	0.12 *
12–17 years (*N* = 250; Free parameters = 30; H0 Loglikelihood = −1886.50; Scaling correction factor for MLR = 1.37; AIC = 3832.99)				
Speed	0.58 **	4.40 **	0.55 **	−0.87 **
Control	0.36 **	2.82 **	0.34 **	−0.53 **
Verbal WM	0.25 **	0.57 **	−0.01	0.10
Visual WM	−0.04	−0.83	−0.25 **	0.28 **
Gf	0.30 **	0.244	0.02	0.01

Note 1: Only nonlinear interactive models are presented here and in all studies following; these models do not produce sample-level covariance expectations and, hence, no absolute fit indices are available; the H0 loglikelihood, the scaling correction factor for the MLR chi-square test statistic and AIC (Akaike’s information criterion) of each model are presented for indicative purposes. Nonlinear models always fit better than linear models; the fit obtained in all of the studies was always acceptable, as suggested by the MLR chi-square test statistic. Note 2: Loadings in this and all other tables following are not standardized. The symbol “–“, whenever appears, indicates that the relation indicated is dropped from the model, because it was very low and caused problems in convergence. Note 3: WM and Gf stand for working memory and fluid intelligence, respectively, wherever they appear in the paper. * *p* < 0.05; ** *p* < 0.005.

**Table 2:** Differentiation models based on the integrated Greek and Chinese sample (*N* = 299).

Ability	Age	*g*	*g*^2^	*g* × age
GR-CHI ALL (*N* = 298; Free parameters = 34; H0 Loglikelihood = −2409.84; Scaling correction factor for MLR = 1.29; AIC = 4903.68)				
Speed	0.35 **	0.62 **	-	−0.18 **
Control	0.16 **	0.78 **	-	−0.26 **
Inhibition	0.27 **	0.74 **	-	−0.24 **
WM	0.33 **	−0.01	-	0.13 **
Deductive	0.29 **	0.04	-	0.05
Inductive	0.46 **	−0.13	-	0.18 **
Awareness-sim	0.37 **	−0.08	-	0.10 **
Awareness-dif	0.33 **	−0.08	-	0.14 **

Note: Awareness-sim and Awareness-dif stand for awareness of similarities between mental processes and differences between mental processes in the mental load they impose, respectively. * *p* < 0.05; ** *p* < 0.005.

**Table 3:** Differentiation models based in the cycle of rule-based thought (*N* = 344).

Ability	Age	*g*	*g*^2^	*g* × age
Whole sample (*N* = 343; Free parameters = 61; H0 Loglikelihood = −6079.32; Scaling correction factor for MLR = 1.06; AIC = 12280.65)				
Speed	0.20	0.66 **	−0.22 **	−0.08
Control	0.07	0.52 **	−0.16 *	0.10
Inhibition	0.11	0.47 **	−0.16 *	0.03
WM	0.13	0.50 **	−0.08	−0.04
Raven A	−0.04	0.48 **	−0.08	0.08
Raven B	0.04	0.62 **	−0.16 *	0.07
Raven C	−0.02	0.59 **	−0.06	0.12 *
Awareness-Perc	0.24	0.37 **	0.13 *	−0.16 *
Awareness-Infr	-	0.52 **	0.31 **	−0.12
Group 5–6 years (*N* = 172; Free parameters = 63; H0 Loglikelihood = −2863.14; Scaling correction factor for MLR = 1.46; AIC = 5852.28)				
Speed	0.43 **	0.29	0.20	−0.42
Control	−0.03	0.49	−0.17	0.07
Inhibition	0.36 **	0.06	−0.04	0.09
WM	0.36 **	0.37 **	0.12	−0.15
Raven A	−0.27	0.46	0.06	−0.08
Raven B	−0.11	0.64	−0.04	0.22
Raven C	−0.26	0.51	−0.01	0.09
Awareness-Perc	0.16	-	0.27 **	−0.48 **
Awareness-Infr	-	0.14	0.46	−0.45
Group 7–11 years (*N* = 171; Free parameters = 63; H0 Loglikelihood = −2828.76; Scaling correction factor for MLR = 1.13; AIC = 5743.51)				
Speed	0.19 **	0.14 *	0.10 *	−0.08
Control	0.24 **	0.07	0.00	0.02
Inhibition	0.21 **	−0.06	0.12 **	−0.01
WM	0.17 **	0.02	0.08	−0.03
Raven A	0.09 **	0.56 **	−0.57 **	0.10 **
Raven B	0.23 **	0.18 **	−0.02	−0.14 **
Raven C	0.27 **	0.23 **	−0.15 **	−0.13
Awareness-Perc	0.22 **	-	0.15 **	−0.18 **
Awareness-Infr	-	0.49 **	0.18 **	−0.21 **

Note: Raven A, B, and C stand for successive difficulty levels of Raven-like matrices. Awareness-Perc and Awareness-Infr stand for perceptual and inferential awareness, respectively. * *p* < .05; ** *p* < .005.

**Table 4:** Differentiation models focusing on inductive and deductive reasoning from 7 to 12 years of age (*N* = 395).

Ability	Age	*g*	*g*^2^	*g* × age
Group 7–12 yrs (*N* = 395; Free parameters = 59; H0 Loglikelihood = −5505.18; Scaling correction factor for MLR = 1.11; AIC = 11,128.37)				
Speed	0.32 **	0.15	−0.05	−0.05
Control	0.38 **	0.36 **	−0.11	−0.11
Conc. Control	0.31 **	0.16	−0.04	−0.04
PWM	0.18 **	0.41 **	−0.10	−0.10
NWM	0.13 **	0.33 **	−0.02	−0.02
NSWM	0.14 **	0.27 **	−0.04	−0.04
Inductive L1	0.37 **	0.41 **	−0.06	−0.06
Inductive L2	0.30 **	0.37 **	−0.01	−0.01
Inductive L3	0.25 **	0.39 **	0.20 **	0.20 **
Deductive L1	0.23 **	0.40 **	−0.06	−0.06
Deductive L2	0.30 **	-	0.05 *	0.05 *
Deductive L3	0.17 **	0.02	0.04	0.04

Note: PWM, NWM, and NSWM stand for perceptual, numeric, and numeric-symbolic working memory, respectively. L1-3 stand for levels 1–3, respectively, of inductive and deductive reasoning batteries. * *p* < 0.05; ** *p* < 0.005.

**Table 5:** Differentiation models focusing on processing efficiency, inductive and mathematical reasoning from 11 to 17 years of age (*N* = 478).

Ability	Age	*g*	*g*^2^	*g* × age
Group 11–17 years (*N* = 289; Free parameters = 62; H0 Loglikelihood = −2404.55; Scaling correction factor for MLR = 1.49; AIC = 4933.10)				
Speed	0.19 **	0.02	−0.01	−0.01
Control	0.12 **	0.07	−0.03	−0.03
Div. Attention	0.12 **	0.10	−0.03	−0.03
BDS	0.21 **	0.25 **	0.04 *	0.04 *
Maths L1	0.10 **	0.21 **	−0.02	−0.02
Maths L2	0.18 **	0.32 **	−0.01	−0.01
Maths L3	0.23 **	0.28 **	0.02 *	0.02 *
Maths L4	0.26 **	0.28 **	0.03 **	0.03 **
Raven L1	0.02 *	0.18 **	−0.06 **	−0.06 **
Raven L2	0.08 **	0.40 **	−0.09 **	−0.09 **
Raven L3	0.15 **	0.45 **	−0.07 **	−0.07 **
Raven L4	0.30 **	0.53 **	0.09 **	0.09 **
Group 11–17 years (*N* = 289; Free parameters = 78; H0 Loglikelihood = −5505.18; Scaling correction factor for MLR = 1.11; AIC = 6356. 59)				
Speed	0.19 **	−0.03	−0.02	0.00
Control	0.13 **	0.01	−0.06	−0.05 *
Div. Attention	0.11 **	0.00	−0.05	−0.04
BDS	0.19 **	0.21 **	0.03	0.04
Prop L1	0.16 **	0.31 **	0.05	0.02
Prop L2	0.26 **	0.43 **	0.15 **	0.16 **
Prop L3	0.29 **	0.32 **	0.18 **	0.21 **
Prop L4	0.29 **	0.37 **	0.21 **	0.21 **
Raven L1	0.01	0.10 **	−0.11 **	−0.03
Raven L2	0.05 **	0.35 **	−0.18 **	−0.07 **
Raven L3	0.12 **	0.33 **	−0.15 **	−0.04 *
Raven L4	0.26 **	0.36 **	0.01	0.05 *

Note: Div. Attention stands for divided attention. BDS stand for backward digit span. L1–4 stand for levels 1–4, respectively. * *p* < 0.05; ** *p* < 0.005.

**Table 6:** Differentiation models focusing on mathematical, causal, propositional, and spatial reasoning and awareness about them from 11 to 20 years of age (*N* = 621).

Ability	Age	*g*	*g*^2^	*g* × age
Metacognitive 11–20 years (*N* = 621; Free Parameters = 54; H0 Loglikelihood = −7139.58; Scaling correction factor for MLR = 1.08; AIC = 14,387.15)				
Causal P	0.19 **	0.44 **	0.02	0.02
Mathematical P	0.19 **	0.58 **	0.04	−0.01
Spatial P	0.16 **	0.58 **	−0.16 **	−10 **
Deductive P	0.14 **	0.37 **	0.18 **	−0.02
Causal SE	0.10 **	0.19 **	0.02	−0.05 *
Mathematical SE	0.12 **	0.48 **	−0.04	−0.05*
Spatial SE	0.10 **	0.41 **	−0.22 **	−0.10 **
Deductive SE	0.09 **	0.25 **	0.15 **	−0.08 **
Cognizance SIM	0.18 **	0.55 **	0.23 **	0.13 **

Note: P stands for actual performance on tasks. SE stands for self-evaluation of performance. Cognizance SIM stands for evaluation of similarities between mental processes in tasks. * *p* < 0.05; ** *p* < 0.005.

**Table 7:** Differentiation models focusing on processing efficiency, working memory and inductive reasoning from 11 to 85 years of age (*N* = 417).

Ability	Age	*g*	*g*^2^	*g* × age
Participants from 11–30 years (*N* = 242; H0 Loglikelihood = −1008.57; Scaling correction factor for MLR = 1.90; AIC = 2101.15)				
Speed	0.00	0.22 **	−0.10 **	0.02
Perceptual Discrimination	0.00	0.24 **	−0.17 **	0.03 **
Attention Control	0.00	0.28 **	−0.08 **	0.01
Conceptual Control	0.00	0.20 **	−0.07 **	0.01
STM	0.01	0.16 *	−0.03	0.02
WM	0.01	0.11	0.02	0.01
Reason Full Test	0.04	0.40 **	−0.01	0.12 *
*Rule-based*	−0.05 **	0.15 **	*0.00*	0.05 **
*Principle-based*	*0.02*	0.48 **	0.22 **	0.06 **
*Adv. principle-based*	−0.05 **	*0.01*	*0.05*	0.04 *
Participants from 31–85 years (*N* = 175; H0 Loglikelihood = −1682.27; Scaling correction factor for MLR = .92; AIC = 3448.54)				
Speed	−0.03 **	0.92 **	−0.17 **	0.02 **
Perceptual Discrimination	−0.03 **	1.10 **	−0.26 **	0.02 **
Att. Control	−0.03 **	0.51 **	−0.07 *	0.01 *
Conc. Control	−0.04 **	0.75 **	−0.08 **	0.01 **
STM	−0.03 **	0.37 **	−0.01	0.00
WM	−0.03 **	0.21 *	−0.16 **	0.00
Reason Full Test	−0.11 **	1.06 **	0.07	−0.02
*Rule-based*	−0.04 **	*-*	0.17 **	*0.00*
*Principle-based*	*−0.01*	0.20 **	0.20 **	*−0.01*
*Adv. principle-based*	*0.00*	*0.08*	*0.00*	*-*

Note: Each panel presents two applications of the model. The first application involved all speeded performance and all working memory scores and the total score on the IRDT. In the second application, the total IRDT score was dropped and the three level-specific scores were involved. Results from this second application are italicized in the table. The Advanced (Adv.) principle-based score stands for performance on post-formal thought, in terms of Commons’s model of Hierarchical complexity. * *p* < 0.05; ** *p* < 0.005.

**Table 8:** Differentiation models focusing on spatial, deductive, and social reasoning, self-evaluation in the respective domains, and general cognitive self-representation about them from 14 to 45 years of age (*N* = 282).

Ability	Age	*g*	*g*^2^	*g* × age
Group 14–45 years (*N* = 282; Free parameters = 62; H0 Loglikelihood = −4162.29; Scaling correction factor for MLR = 1.25; AIC = 8448.58)				
Spatial P	−0.06	0.24 **	−0.51 **	0.04
Deductive P	−0.08	0.18 **	−0.24 **	0.04
Social P	0.38 **	0.10	0.02	0.03
Spatial SE	0.08	0.59 **	−0.72 **	0.09
Deductive SE	0.12 *	0.36 **	−0.31 **	0.00
Social SE	0.20 **	0.31 **	−0.18 *	0.03
Spatial SR	−0.11	0.57 **	0.00	−0.08
Deductive SR	−0.09	0.44 **	0.23 **	0.00
Social SR	0.15 **	0.26 **	0.04	−0.07
Representational efficiency SR	−0.08	0.48 **	0.22 **	−0.07

Note: P stands for actual performance on tasks. SE stands for self-evaluation of performance on tasks. SR stand for general self-representation of ability in the domain concerned. * *p* < 0.05; ** *p* < 0.005.

## Supplementary Materials

The following are available online at http://www.mdpi.com/2079-3200/5/2/23/s1, Tables S1–S8: Correlation Matrices in the Models Tested in Studies 1–8.
