# Supplementary material for: Developmental Differentiation and Binding of Mental Processes with g through the Life-Span
Source: J Intell. 2017 May 31;5(2):23. doi: 10.3390/jintelligence5020023 (PMC6526403; doi:10.3390/jintelligence5020023)
Supplement: Supplementary file 1 [file jintelligence-05-00023-s001.pdf]

Journal of Intelligence

Table S1. Correlation Matrices in the Models Tested in Study 1.

	1	2	3	4	5	6	7	8
1. Age	1							
2. *g* factor score	.661 **	1						
3. *g* × age	.693 **	.943 **	1					
4. Speed	.606 **	.212 **	.311 **	1				
5. Control	.577 **	.244 **	.343 **	.872 **	1			
6. Verbal WM	.447 **	.335 **	.359 **	.354 **	.328 **	1		
7. Visual WM	.577 **	.951 **	.926 **	.184 **	.214 **	.296 **	1	
8. Gf	.872 **	.770 **	.768 **	.510 **	.490 **	.469 **	.643 **	1

* $p < .05$, ** $p < .01$.

Table S2. Correlation Matrices in the Models Tested in Study 2.

	1	2	3	4	5	6	7	8	9	10	11
1. Age	1										
2. *g* factor score	.758 **	1									
3. *g* × age	.795 **	.985 **	1								
4. Speed	.395 **	.137 *	.197 **	1							
5. Control	.143 *	−.056	.003	.687 **	1						
6. Inhibition	.287 **	.089	.146 *	.730 **	.856 **	1					
7. WM	.575 **	.624 **	.624 **	.112	−.035	.105	1				
8. Deductive	.361 **	.409 **	.411 **	.219 **	.066	.091	.292 **	1			
9. Inductive	.588 **	.766 **	.741 **	.063	−.132 *	−.001	.531 **	.321 **	1		
10. Awareness-sim	.509 **	.779 **	.761 **	.058	−.052	.042	.474 **	.349 **	.563 **	1	
11. Awareness-dif	.490 **	.746 **	.734 **	.046	−.069	.013	.398 **	.232 **	.480 **	.505 **	1

* $p < .05$, ** $p < .01$.

Table S3. Correlation Matrices in the Models Tested in Study 3.

	1	2	3	4	5	6	7	8	9	10	11	12
1. Age	1											
2. *g* factor score	.834 **	1										
3. *g* × age	.852 **	.970 **	1									
4. Speed	.596 **	.759 **	.703 **	1								
5. Control	.497 **	.635 **	.622 **	.374 **	1							
6. Inhibition	.495 **	.600 **	.585 **	.408 **	.334 **	1						
7. WM	.576 **	.631 **	.633 **	.350 **	.350 **	.273 **	1					
8. Raven A	.393 **	.459 **	.458 **	.279 **	.218 **	.059	.239 **	1				
9. Raven B	.538 **	.645 **	.626 **	.428 **	.355 **	.237 **	.373 **	.439 **	1			
10. Raven C	.511 **	.591 **	.611 **	.389 **	.324 **	.169 **	.401 **	.406 **	.543 **	1		
11. Awareness-Perc	.386 **	.466 **	.448 **	.361 **	.170 **	.185 **	.330 **	.183 **	.226 **	.193 **	1	
12. Awareness-Infr	.411 **	.541 **	.548 **	.356 **	.293 **	.195 **	.336 **	.274 **	.319 **	.316 **	.302 **	1

* $p < .05$, ** $p < .01$.

Table S4. Correlation Matrices in the Models Tested in Study 4.

	1	2	3	4	5	6	7	8	9	10	11	12	13	14	15
1. Age	1														
2. *g* factor score	.648 **	1													
3. *g* × age	.632 **	.983 **	1												
4. Speed	.563 **	.370 **	.351 **	1											
5. Control	.694 **	.641 **	.611 **	.560 **	1										
6. Conc. Control	.552 **	.344 **	.346 **	.589 **	.546 **	1									
7. PWMC	.081	.160 **	.154 **	.003	.093	.023	1								
8. NWMC	.255 **	.342 **	.337 **	.200 **	.278 **	.142 **	.092	1							
9. NSWMC	.271 **	.283 **	.284 **	.204 **	.272 **	.209 **	.089	.458 **	1						
10. Inductive L1	.688 **	.789 **	.757 **	.421 **	.611 **	.407 **	.070	.312 **	.309 **	1					
11. Inductive L2	.566 **	.657 **	.659 **	.409 **	.512 **	.367 **	.057	.286 **	.277 **	.614 **	1				
12. Inductive L3	.493 **	.591 **	.628 **	.277 **	.406 **	.283 **	.039	.250 **	.182 **	.446 **	.404 **	1			
13. Deductive L1	.439 **	.750 **	.734 **	.297 **	.410 **	.292 **	.136**	.245 **	.249 **	.522 **	.370 **	.328 **	1		

14. Deductive L2	.523 **	.683 **	.683 **	.330 **	.505 **	.349 **	.070	.273 **	.241 **	.550 **	.461 **	.414 **	.446 **	1	
15. Deductive L3	.296 **	.042	.038	.219 **	.218 **	.233 **	.019	.143 **	.135 **	.236 **	.224 **	.186 **	.077	.243 **	1

* $p < .05$, ** $p < .01$.

Table S5. Correlation Matrices in the Models Tested in Study 5.

	**1**	**2**	**3**	**4**	**5**	**6**	**7**	**8**	**9**	**10**	**11**	**12**	**13**	**14**	**15**
1. Age	1														
2. *g* factor score	.810 **	1													
3. *g* × age	.843 **	.990 **	1												
4. Speed	.566 **	.670 **	.644 **	1											
5. Control	.427 **	.480 **	.454 **	.453 **	1										
6. Div. Attention	.608 **	.624 **	.605 **	.534 **	.660 **	1									
7. BDS	.492 **	.742 **	.745 **	.346 **	.256 **	.317 **	1								
8. Maths L1	.516 **	.609 **	.599 **	.370 **	.365 **	.441 **	.384 **	1							
9. Maths L2	.704 **	.789 **	.791 **	.404 **	.347 **	.516 **	.498 **	.684 **	1						
10. Maths L3	.773 **	.848 **	.857 **	.463 **	.448 **	.565 **	.529 **	.598 **	.836 **	1					
11. Maths L4	.800 **	.854 **	.872 **	.468 **	.344 **	.524 **	.526 **	.591 **	.834 **	.858 **	1				
12. Raven L1	.122 *	.190 **	.186 **	.120 *	.159 **	.121 *	.132 *	.333 **	.291 **	.207 **	.201 **	1			
13. Raven L2	.335 **	.395 **	.398 **	.206 **	.228 **	.308 **	.289 **	.482 **	.589 **	.494 **	.460 **	.406 **	1		
14. Raven L3	.497 **	.551 **	.557 **	.286 **	.283 **	.430 **	.376 **	.649 **	.692 **	.606 **	.617 **	.361 **	.683 **	1	
15. Raven L4	.683 **	.763 **	.783 **	.374 **	.322 **	.461 **	.532 **	.547 **	.741 **	.796 **	.770 **	.189 **	.489 **	.642 **	1

* $p < .05$, ** $p < .01$.

Table S6. Correlation Matrices in the Models Tested in Study 6.

	1	2	3	4	5	6	7	8	9	10	11	12
1. Grade	1											
2. g factor score	.466 **	1										
3. g χ age	.342 **	.839 **	1									
4. Causal P	.412 **	.568 **	.453 **	1								
5. Mathematical P	.422 **	.813 **	.677 **	.462 **	1							
6. Spatial P	.350 **	.772 **	.650 **	.410 **	.495 **	1						
7. Deductive P	.338 **	.306 **	.166 **	.414 **	.368 **	.267 **	1					
8. Causal SE	.209 **	.264 **	.267 **	.385 **	.175 **	.133 **	.160 **	1				
9. Mathematical SE	.277 **	.718 **	.611 **	.272 **	.649 **	.339 **	.200 **	.217 **	1			
10. Spatial SE	.208 **	.577 **	.554 **	.182 **	.228 **	.469 **	.135 **	.135 **	.229 **	1		
11. Deductive SE	.215 **	.123 **	.060	.255 **	.198 **	.140 **	.593 **	.227 **	.166 **	.137 **	1	
12. Cognizance SIM	.414 **	.572 **	.328 **	.418 **	.468 **	.327 **	.378 **	.140 **	.309 **	.181 **	.203 **	1

* $p < .05$, ** $p < .01$.

Table S7. Correlation Matrices in the Models Tested in study 7.

	1	2	3	4	5	6	7	8	9	10
1. Age	1									
2. *g* factor score	−.068	1								
3. *g* × age	−.156 **	.818 **	1							
4. Speed	−.660 **	.586 **	.729 **	1						
5. Perceptual Discrimination	−.604 **	.652 **	.785 **	.902 **	1					
6. Att. Control	−.735 **	.537 **	.614 **	.768 **	.772 **	1				
7. Conc. Control	−.700 **	.562 **	.694 **	.826 **	.831 **	.809 **	1			
8. STM	−.630 **	.350 **	.423 **	.642 **	.600 **	.649 **	.661 **	1		
9. WM	−.375 **	.174 **	.217 **	.391 **	.363 **	.374 **	.368 **	.552 **	1	
10. Reason Full Test	−.704 **	.261 **	.308 **	.612 **	.598 **	.647 **	.652 **	.590 **	.307 **	1

* $p < .05$, ** $p < .01$.

Table S8. Correlation Matrices in the Models Tested in Study 8.

	1	2	3	4	5	6	7	8	9	10	11	12	13
1. Age	1												
2. *g* factor score	.033	1											
3. *g* × age	.033	.936 **	1										
4. Spatial R	−.033	.675 **	.639 **	1									
5. Deductive R	−.062	.136 *	.133 *	.379 **	1								
6. Social R	.397 **	.001	.012	.108	.115	1							
7. Spatial SE	.130 *	.905 **	.858 **	.484 **	.234 **	.068	1						
8. Deductive SE	.155 **	.310 **	.323 **	.260 **	.287 **	.086	.414 **	1					
9. Social SE	.232 **	.213 **	.254 **	.091	.082	.320 **	.320 **	.426 **	1				
10. Spatial SR	−.087	.246 **	.222 **	.126 *	.119 *	−.012	.289 **	.191 **	.169 **	1			
11. Deductive SR	−.074	.033	.021	−.041	.018	.052	.042	.023	.116	.453 **	1		
12. Social SR	.169 **	−.114	−.101	.014	.156 **	.230 **	.085	.152 *	.217 **	.349 **	.212 **	1	
13. Representational efficiency SR	−.064	.029	.026	−.064	.005	.059	.058	.125 *	.082	.474 **	.504 **	.190 **	1

* $p < .05$, ** $p < .01$.
